# Impact of non-pharmaceutical interventions against COVID-19 in Europe in 2020: a quasi-experimental non-equivalent group and time series design study

**DOI:** 10.2807/1560-7917.ES.2021.26.28.2001401

**Published:** 2021-07-15

**Authors:** Paul R Hunter, Felipe J Colón-González, Julii Brainard, Steven Rushton

**Affiliations:** 1 Norwich Medical School, University of East Anglia, Norwich, United Kingdom; 2 Department of Environmental Health, Tshwane University of Technology, Pretoria, South Africa; 3 Department of Infectious Disease Epidemiology, London School of Hygiene and Tropical Medicine, London, United Kingdom; 4 School of Environmental Sciences, University of East Anglia, Norwich, United Kingdom; 5 Tyndall Centre for Climate Change Research, University of East Anglia, Norwich, United Kingdom; 6 School of Natural and Environmental Sciences, Newcastle University, Newcastle, United Kingdom

**Keywords:** COVID-19, control measures, stay at home, collinearity, Bayesian generalised additive mixed models

## Abstract

**Introduction:**

The current pandemic of coronavirus disease (COVID-19) is unparalleled in recent history as are the social distancing interventions that have led to a considerable halt on the economic and social life of so many countries.

**Aim:**

We aimed to generate empirical evidence about which social distancing measures had the most impact in reducing case counts and mortality.

**Methods:**

We report a quasi-experimental (observational) study of the impact of various interventions for control of the outbreak through 24 April 2020. Chronological data on case numbers and deaths were taken from the daily published figures by the European Centre for Disease Prevention and Control and dates of initiation of various control strategies from the Institute of Health Metrics and Evaluation website and published sources. Our complementary analyses were modelled in R using Bayesian generalised additive mixed models and in STATA using multilevel mixed-effects regression models.

**Results:**

From both sets of modelling, we found that closure of education facilities, prohibiting mass gatherings and closure of some non-essential businesses were associated with reduced incidence whereas stay-at-home orders and closure of additional non-essential businesses was not associated with any independent additional impact.

**Conclusions:**

Our findings are that schools and some non-essential businesses operating ‘as normal’ as well as allowing mass gatherings were incompatible with suppressing disease spread. Closure of all businesses and stay at home orders are less likely to be required to keep disease incidence low. Our results help identify what were the most effective non-pharmaceutical interventions in this period.

## Introduction

The current pandemic of coronavirus disease (COVID-19) is unprecedented in modern history. Not only is the impact of the epidemic being measured by the number of cases and deaths, but also by its impact on overloaded health services and undesirable impacts on quality of life and near-future economic prospects. Wider society was subjected at times to an almost complete stasis of social and cultural life. The benefits of social distancing was shown earliest in China, Italy and Spain that turned the tide on their country’s epidemics using often severe social distancing strategies. These examples do not indicate the relative importance of the different non-pharmaceutical/social distancing interventions. Given the potentially high economic and social costs arising from stringent control measures [1-5], it has been imperative to determine which social distancing measures are most effective at controlling the pandemic. Imposition and relaxation of control measures should be informed by such knowledge. Early on in pandemic response, much policy was driven by the results of mathematical models [6]. However, there was much concurrent public debate about the validity and limitations of the different models for policy making and modelling approaches that were used [7-10]. It is also useful to assess empirical evidence of what aspects of currently applied non-pharmaceutical interventions (NPI) have or have not been effective.

A quasi-experimental study design is an observational study where the allocation to receive the intervention (or not) is not randomly made [11,12]. Most European states introduced a similar suite of interventions aimed at reducing contact between individuals to reduce transmission. The different types of intervention used and their timing varied from one country to another and arose in response to political processes in each country. No measure that we will consider in this analysis was imposed by all European countries. Where measures were put in place, they were often imposed at different points in the development of the epidemics. By late April 2020, some European countries were easing control measures so late April was a good point to take stock of intervention effects. This situation offered a unique opportunity to investigate the putative impacts of the various types of intervention, as each epidemic in an individual country forms what is effectively a chrono-sequence of disease spread. The intervention strategies could then be compared as interrupted time series.

We report here analyses of trends in both reported cases and deaths across 30 European countries with rather different approaches to and timing of restrictions. We use a quasi-experimental approach to identify what affects such restrictions may have had on the control of the epidemic.

## Methods

### Data

Data on new cases and deaths reported by all countries were obtained from the European Centre for Disease Prevention and Control (https://www.ecdc.europa.eu/en/publications-data/download-todays-data-geographic-distribution-covid-19-cases-worldwide ). Data up to 24 April 2020 are included. For the United Kingdom (UK), we used only the so-called pillar 1 case numbers. Pillar 1 refer to swab testing in Public Health England laboratories and National Health Service hospitals for those with a clinical need, and for health and care workers. Pillar 2 results (rt-PCR testing for persons with suspected COVID-19 in the wider community) as reported daily on the UK government coronavirus data website (https://www.gov.uk/guidance/coronavirus-covid-19-information-for-the-public#history) were removed from the case numbers, as pillar 2 sampling was only introduced late in the course of the UK epidemic and inflated total case numbers relative to earlier in the UK outbreak. We also adjusted our results by the number of tests reported per 1 million population, taken on 16 April from WorldoMeter (https://www.worldometers.info/coronavirus/). In order to compare time series for different countries with different dates of onset for their own epidemics we chose to define the onset as the first day when a case was reported after the latest time where there were two or more consecutive days with no cases reported.

The dates when (if at all) each of the various social restrictions were imposed in the 30 European countries included in this analysis were given by the Institute of Health Metrics and Evaluation Data (IHME) (https://covid19.healthdata.org/). The six categories of restrictions were ‘mass gathering restrictions’, ‘initial business closure’, ‘educational facilities closed’, ‘non-essential services closed’, ‘stay-at-home order’ and ‘travel severely limited’. However, no country was listed in the dataset as having severe travel restrictions during the monitoring period so we dropped this category from any further analysis. The IHME definitions of these measures are given on their website. We paraphrase the definitions here:

- Mass gathering restrictions were mandatory restrictions on private or public gatherings of any number of people.
- Initial business closure refers to the first time that there was any mandatory closure of businesses, not necessarily all businesses. Usually such initial closures would primarily affect businesses such as entertainment venues, bars and restaurants.
- Where non-essential businesses were ordered to close, this usually included many more businesses than were in the first closure category. The second wave of closures probably included general retail stores and services such as hairdressers.
- Closure of education facilities included all levels of education (primary, secondary and higher) that stopped face-to-face teacher-to-student teaching.
- Stay-at-home orders affected all individuals unless travelling for essential services. They allowed close contact only with people of the same household and perhaps some outdoors exercise.

In three countries (Germany, Italy and Spain), the restrictions were not implemented uniformly through the country on precisely the same dates so we took the median date for the nation; the actual variations in dates were extremely small in Italy and Spain and only somewhat diverse in Germany (see Supplement 1, part 1). Among the 16 German states, 15 states imposed mass gathering restrictions within 2 days of the median date used, nine states had initial business closures within 2 days of the median date, 15 states closed educational establishments within 2 days of the median date, nine states closed non-essential businesses within 2 days of the median German date and all states imposed stay-at-home orders within 2 days of the median national date. 

All models adjusted for when countries started to advise or mandate their citizens to wear face masks or coverings (dates of face cover measures are listed in Supplement 1, part 2). We included when countries either mandated or encouraged the wearing of face coverings or masks in public places as an independent control measure in the models. However, it was obvious that how such advisories or mandates were implemented varied considerably from one country to another. For example, in some countries, face masks were required both outdoors and indoors in public and in others only in indoor settings. Sometimes, mask wearing was required in few settings such as on public transport, other times in several settings such as on public transport, in shops and in schools. Also mask-wearing mandates, where implemented, were only introduced relatively late in the monitoring period, often even as other control measures were being relaxed, which complicated interpretation of how much masks may have helped reduce transmission. Consequently, although we included the wearing of face coverings in the analyses, we caution against drawing any strong conclusions over their value based on these analyses alone. 

### Analyses

We undertook two sets of analyses. In order to ensure comparability between countries with different timing of their outbreaks we counted dates as being from the start, the epidemic in each country was assumed to have commenced on the first day with a reported case after the last time that no cases were reported on two consecutive days.

The first analysis was done in R using Bayesian generalised additive mixed-effects models. These incorporate both fixed and random effects (i.e. mixed effects) to adjust for spatial dependency in disease between nation states. Random effects correspond to those for which levels are samples from a larger population, whereas fixed effects correspond to average effects for the whole population. Examples of fixed effects would be interventions such as shutting all schools and making people work at home. Other sources of variation that contribute may be more random and associated with unmeasured features of the sampling unit (the nation state). Key here is the fact that the nation states differ culturally and in other features such as recording methods. We have not measured the source of the variation but we know it is associated with the sampling unit (state) with which the response is recorded through time. In addition, we also anticipated spatial effects because most European states experiencing COVID-19 epidemics had porous land borders under the Schengen Area agreement. We therefore expect some spatial dependency between states as the closer they are to each other the more likely it is that they have similar patterns of disease. Bayesian models are very useful as they allow us to quantify the relative contributions of fixed, random, temporal and spatial dependency in the same modelling framework.

The variance in the COVID-19 data was four orders of magnitude larger than the mean number of cases and three orders of magnitude larger than the mean number of deaths. Consequently, models were fit using a negative binomial specification to account for potential over-dispersion in the data, and within a conditional autoregressive model (Besag–York–Mollié) framework [13] to allow for potential spatial autocorrelation and unstructured between-country variation.

Let *Yi*,*t* be the number of COVID-19 cases or deaths for country *i* = 1, ⋯, *I* at time *t* = 1, ⋯, *T.* The general algebraic definition of the models is given by:

*Y_i_*_,_ *_t_*∣*μ_i_*_,_ *_t_*,*ϕ*∼*NegBin*(*μ_i_*_,_ *_t_*,*ϕ*),

where *Y_i_*_,_ *_t_* is the number of COVID-19 cases or deaths for country *i* = 1, ⋯, *I* at time *t* = 1, ⋯, *μ_i_*_,_ *_t_* is the predicted number of COVID-19 cases or deaths for country *i* and time *t*, and *ϕ* > 0 is the negative binomial dispersion parameter. A logarithmic link function of the expected number of cases or deaths was modelled as:

$$log(\mu i,t)=\;\alpha \;+\;log(Pi,d[t])\;+\;\delta Di,d[t]\;+\;\epsilon Ri,d[t]\;+\sum_{k}{\beta X}_{i,t,k}+\;ui\;+\;\nu i,$$

where *α* corresponded to the intercept; log(*P_i_*_,_ *_d_*_[_ *_t_*_]_) denotes the logarithm of the population at risk for country *i* and day *d*_[_ *_t_*_]_ was included as an offset to adjust case counts by population. *D_i_*_,_ *_d_*_[_ *_t_*_]_ is a linear term for the number of days since the outbreak started, with coefficient *δ. R_i_*_,_ *_d_*_[_ *_t]_* is a linear function of the number of COVID-19 tests carried out per country *i* at day *d*_[_ *_t_*_]_, with regression coefficient *ϵ*. *X* is a matrix of *k* intervention measures (e.g. school and business closures) with regression coefficients *β.* Intervention measures comprise of an index of 1, ⋯,*N* number of days following the intervention being implemented (day 1 was the day following implementation of the intervention). We assumed that the imposition of each intervention led to cumulative changes in effect. Intervention measures were included in the model as a random effect to account for potential nonlinearities in the exposure–response relationship. A random effect adjustment was appropriate because the observation data (case counts) were samples from a larger population (because of limited testing to confirm symptomatic cases and possible asymptomatic cases). Unknown confounding factors with spatial dependency that represent, for example, human mobility, were incorporated using spatially correlated (i.e. structured) random effects (*u_i_*) and independent, identical and normal distributed (i.e. unstructured) random effects (*ν_i_*) for each country *i*. Spatial random effects were specified using a Besag–York–Mollie model to account for spatial dependencies and unstructured variation between countries [14]. Goodness of fit was evaluated using the deviance information criterion (DIC). Models were fitted in R version 3.6.1 using the INLA package.

**Figure 1:**
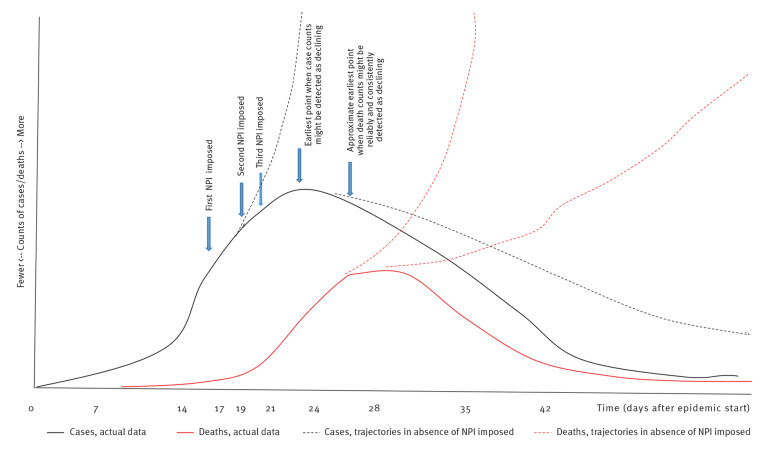
Exemplaric timeline of possible non-pharmaceutical intervention impositions and potential epidemic response, COVID-19 pandemic, Europe, 2020

COVID-19: coronavirus disease; NPI: non-pharmaceutical intervention.

The second analysis was a multilevel mixed-effects regression analysis in STATA v 16.1 (StataCorp LLC, College Station, United States). We used a mixed-effects negative binomial regression model with cases or deaths on a specific day as the outcome variable, country population as the exposure variable, country as a mixed effect and days from start of the epidemic as a fixed effect. Fixed effect was appropriate for days elapsed because we were looking for possible effect of NPI relevant to a fixed start point and over the entire population. All main interventions were included as categorical variables with the week number included as a linear variable after the start of the intervention. Monitoring by week number was appropriate with regard to case counts, given that the incubation period tends to be ca 5 days [15-18] and a small lag between symptom onset and obtaining test results is likely: thus, total days elapsed from exposure to changes in recorded case counts has tended to be ca 7 days. A lag from symptom onset to hospitalisation of ca 7 days [19,20] and a similar subsequent lag (ca 7 days) from hospitalisation to death are reported in COVID-19 literature [19-21]. Figure 1 indicates the impact of key likely onsets of intervention on an exemplaric epidemic curve. For simplicity and brevity we report only on the results for the 7-day categorisation in this manuscript. However, in view of the variation in incubation period and the possibility that this might have interfered with the parameter estimates, we repeated Analysis 2 for three alternative response time periods (post-intervention) as sensitivity analyses. These alternative response periods were 4 days, 10 days and 14 days. The resulting incident risk ratios (between our preferred response period of 7 days and alternatives) could then be compared for possible trend differences. In further sensitivity and collinearity checks, we dropped each of the main predictor variables (intervention timings) from the final equation and noted if the regression parameter and standard errors of remaining predictor variables changed dramatically or if the coefficients reversed trend (e.g. went from suggesting increase to suggesting decrease).

The exemplaric timeline shows how epidemic measures (counts of cases or deaths) might respond in the case of three NPI imposed. The ‘earliest point’ refers to the first hypothesised time point in the example, when the epidemic might have been expected to deviate from the trajectory(ies) if no NPI had been imposed.

We also checked for collinearity between the predictor variables by calculating the variance inflation factors (VIF) for the predictors and by calculating the condition number using the coldiag2 command in STATA. A VIF < 10 suggests that model predictors do not have multi-collinearity problems. Values of VIF > 10.0 need to be considered for potential multi-collinearity with regard to other model diagnostics such as condition index and eigenvalues. A condition number > 15 with any variance proportions above 0.9, or if eigenvalues were < 0.01, could suggest collinearity that undermines confidence in coefficient estimates, according to guidance in Chatterjee and Hadi [22] and Regorz [23]. In addition, as sensitivity analysis within Analysis 2, we reran the model dropping each predictor variable in turn to determine whether or not the regression parameters and their standard errors were changed substantially.

### Ethical statement

Ethical approval was not required because this was an analysis of data in the public domain.

## Results

Table 1 lists the estimated date of the start of the epidemic in each country and when each of the five intervention types were implemented, according to the IHME website. ‘Mass gathering restrictions’, ‘initial business closure’, ‘educational facilities closed’, ‘non-essential services closed’ and ‘stay at home order’ were respectively implemented by 29, 28, 29, 23 and 19 countries. Italy was the first country to enter the epidemic on 22 February 2020 and Lithuania the last on 14 March 2020. By our criteria, half of all countries had their epidemic start on or before 27 February.

**Table 1:** Timing of estimated start of each country’s main COVID-19 epidemic and the introduction of social distancing measures, 30 European countries, 2020

Country	Start of main epidemic	Mass gathering restrictions	Initial business closure	Educational facilities closed	Non-essential services closed	Stay at home order	Face covering encouraged or compulsory
Austria	26 Feb	10 Mar	16 Mar	16 Mar	16 Mar	16 Mar	6 Apr
Belgium	2 Mar	13 Mar	13 Mar	14 Mar	18 Mar	18 Mar	NA
Bulgaria	12 Mar	13 Mar	13 Mar	13 Mar	13 Mar	17 Mar	30 Mar
Croatia	11 Mar	9 Mar	19 Mar	16 Mar	19 Mar	17 Mar	NA
Cyprus	10 Mar	24 Mar	24 Mar	13 Mar	24 Mar	24 Mar	NA
Czechia	2 Mar	10 Mar	10 Mar	10 Mar	14 Mar	16 Mar	18 Mar
Denmark	27 Feb	18 Mar	18 Mar	16 Mar	NA	NA	NA
Estonia	11 Mar	13 Mar	13 Mar	16 Mar	NA	NA	5 Apr
Finland	27 Feb	12 Mar	18 Mar	18 Mar	4 Apr	NA	NA
France	26 Feb	4 Mar	14 Mar	12 Mar	14 Mar	16 Mar	5 Apr
Germany	26 Feb	22 Mar	17 Mar	16 Mar	23 Mar	22 Mar	1 Apr
Greece	5 Mar	8 Mar	12 Mar	11 Mar	22 Mar	23 Mar	NA
Hungary	5 Mar	12 Mar	12 Mar	16 Mar	16 Mar	28 Mar	NA
Ireland	4 Mar	12 Mar	15 Mar	12 Mar	24 Mar	27 Mar	NA
Italy	22 Feb	11 Mar	11 Feb	5 Mar	11 Mar	11 Mar	6 Apr
Latvia	8 Mar	13 Mar	NA	12 Mar	NA	NA	NA
Lithuania	14 Mar	15 Mar	14 Mar	16 Mar	15 Mar	15 Mar	1 Apr
Luxembourg	7 Mar	13 Mar	18 Mar	16 Mar	18 Mar	NA	20 Apr
Malta	8 Mar	NA	17 Mar	13 Mar	23 Mar	NA	NA
Netherlands	28 Feb	10 Mar	21 Mar	15 Mar	NA	NA	NA
Norway	27 Feb	12 Mar	12 Mar	12 Mar	NA	NA	5 Apr
Poland	7 Mar	10 Mar	31 Mar	12 Mar	NA	24 Mar	NA
Portugal	3 Mar	19 Mar	16 Mar	16 Mar	19 Mar	19 Mar	16 Apr
Romania	4 Mar	6 Mar	21 Mar	11 Mar	21 Mar	23 Mar	NA
Slovakia	7 Mar	12 Mar	16 Mar	12 Mar	16 Mar	NA	14 Mar
Slovenia	5 Mar	12 Mar	15 Mar	16 Mar	15 Mar	20 Mar	29 Mar
Spain	25 Feb	15 Mar	15 Mar	14 Mar	15 Mar	15 Mar	13 Apr
Sweden	27 Feb	11 Mar	NA	NA	NA	NA	NA
Switzerland	26 Feb	28 Feb	16 Mar	13 Mar	16 Mar	NA	NA
United Kingdom	28 Feb	23 Mar	20 Mar	23 Mar	24 Mar	23 Mar	NA

NA: not applicable, as this control was not implemented; COVID-19: coronavirus disease.

### Analysis 1

Model metrics are presented in Table 2. The dispersion parameter evaluates whether the model is able to cope with potential dispersion in the data. When the value is close to 1 (as it is here) the model is shown to do well at accounting for dispersion.

**Table 2:** Model metrics, impact of non-pharmaceutical interventions, COVID-19 pandemic, Europe, 2020

Model	Deviance information criterion	Watanabe–Akaike information criterion	Conditional predictive ordinate	Dispersion
Cases	18,009.4	18,012.6	−9,006.6	1.01
Deaths	8,032.4	8,035.9	−4,018.4	0.89

COVID-19: coronavirus disease.

The Watanabe–Akaike information criterion (W-AIC) is described by Watanabe in 2010 [46] and was developed to specifically help identify best model fit in Bayesian models. Smaller W-AIC values mean better fit compared with alternative model specifications. The conditional predictive ordinate is a Bayesian diagnostic that detects surprising observations [47].

**Figure 2:**
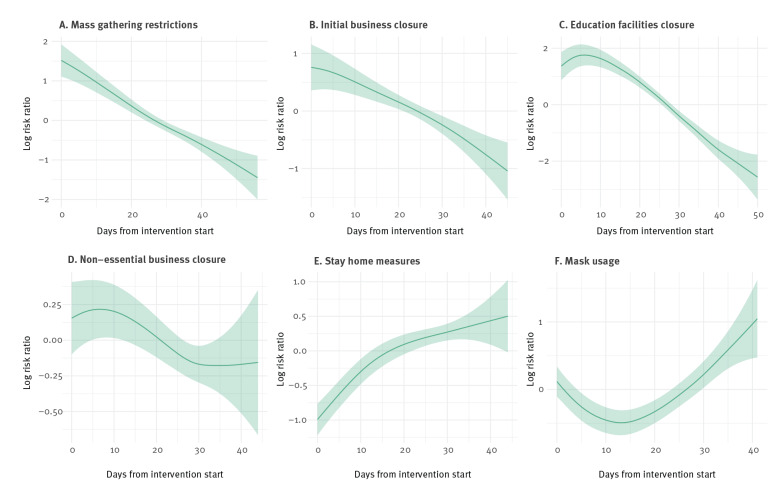
Incidence rate ratios (cases) following implementation of country-level, non-pharmaceutical control measures and daily reported COVID-19 case numbers, 30 European countries, 2020

**Figure 3:**
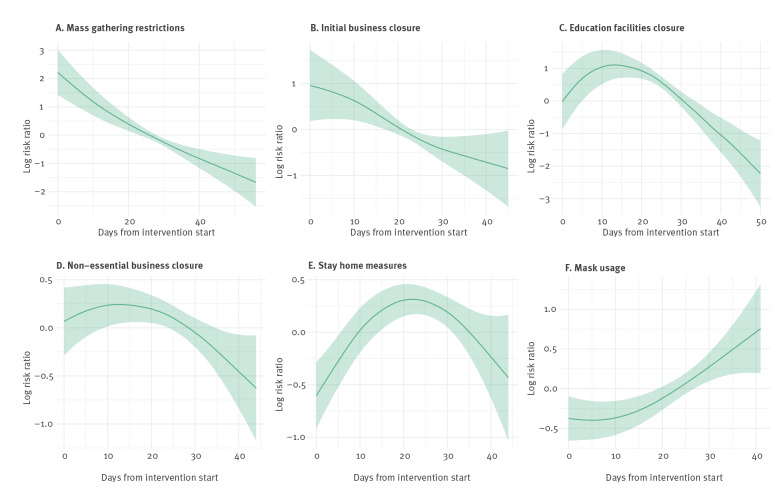
Incidence rate ratios (deaths) following implementation of country-level, non-pharmaceutical control measures and daily reported deaths from COVID-19, 30 European countries, 2020

The exposure–response relationships estimated by the models are presented in Figures 2 (cases) and Figure 3 (deaths). The x-axes represent the days since the intervention started and the y-axes indicate the logarithm of the risk ratio. It can be observed that mass gathering restrictions had a negative effect on the number of cases, with fewer cases occurring as the number of days since intervention started increased. We observed a similar effect for the initial closure of businesses and the closure of education facilities, with less cases occurring as the number of days since the intervention increased. The closure of non-essential businesses did not appear to have a significant effect on the number of COVID-19 cases. This was evident as the estimated relationship and its 95% credible interval stayed close to zero on the y-axis. Surprisingly, stay-at-home measures showed a positive association with cases. This suggests that, as the number of lockdown days increased, so did the number of cases. Negative associations with deaths (Figure 3) were estimated for mass gatherings, initial business closure and the closure of educational facilities, while a non-significant effect was estimated for non-essential business closure. The stay-at-home measures showed an inverted U-quadratic effect with an initial rise of deaths up to Day 20 of the intervention, followed by a decrease. These results suggest that stay-at-home orders may not be required to ensure outbreak control and reduce outbreak harms, provided that all the other control measures are implemented. Of course, if stay-at-home measures are implemented then all the other measures such as business closures, banning mass gatherings and school closures would also follow.

COVID-19: coronavirus disease.

The central line is the posterior mean of the exposure-response relationship; shading is a 95% credible interval.

COVID-19: coronavirus disease.

The central line is the posterior mean of the exposure-response relationship; shading is a 95% credible interval.

The patterns seen in Figures 2 and 3 fit with the understood disease incubation, development and concurrent ascertainment processes. The median incubation period is understood to be 4–7 days [15-17], while case ascertainment tended to require an elapse of 2–10 more days [24]. For severe cases (those who are hospitalised), 8–14 days post symptom onset tends to coincide with the start of a 5–7-day period of peak disease severity [20]. As a result, we expect no intervention should be cited as affecting case counts in under about 7 days, and no intervention is likely to strongly reduce counts of death in less than 2–3 weeks.

For cases and deaths, mask wearing mandates/advisories seem to have initial effects which were either negative (case) or neutral (deaths), followed by rises (in cases or deaths). The overall effect is small compared to other measures, which we confirmed with further sensitivity analyses shown below. The additional benefit of mask-wearing advisories/mandates to the other outbreak control measures seemed to be small and inconsistent. However, for the reasons discussed above we hesitate to interpret these results as certain effects of face cover/mask mandates/advisories.

**Figure 4:**
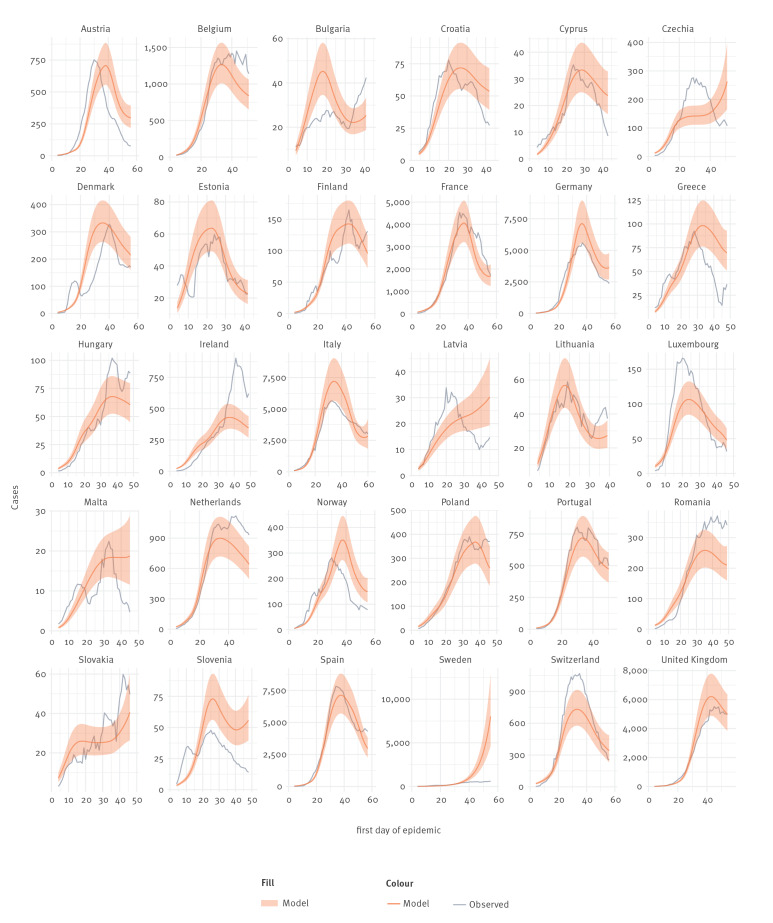
Comparison of predicted daily reports of COVID-19 case numbers with 7-day rolling average actual numbers, 30 European countries, 2020

**Figure 5:**
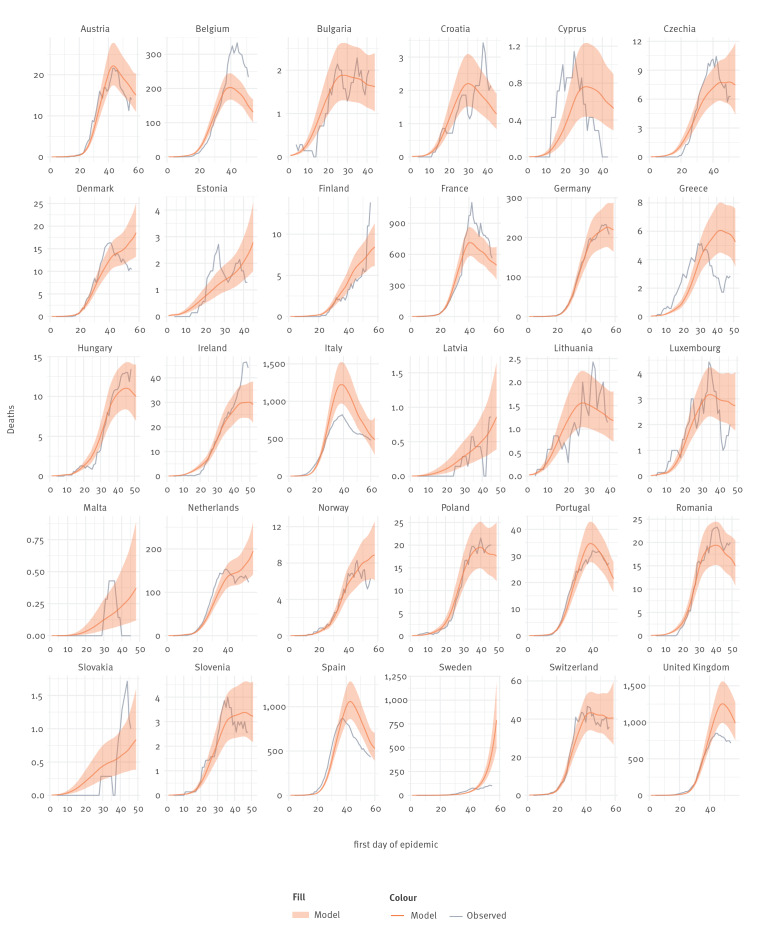
Comparison of predicted daily numbers of reports of deaths from COVID-19 with 7-day rolling average actual numbers, 30 European countries, 2020

Figures 4 and 5 show the association between actual cases and deaths in each country, expressed as 7-day rolling means, and the numbers predicted by the models on cases and deaths. Although for many countries there is a reasonable correlation between the two, this is not the case for all countries and particularly countries with smaller populations. The model outputs especially did not fit Sweden which had much lower numbers of cases and deaths than predicted. This could be explained by partial implementation of controls and unmandated behavioural change in the population. We acknowledge that, at least for some countries, our model could not capture all the temporally changing variables influencing the spread of the disease.

COVID-19: coronavirus disease.

The central line is the posterior mean of the predictions made by the models (for individual countries over time); shading is the 95% credible interval.

COVID-19: coronavirus disease.

The central line is the posterior mean of the predictions made by the models (for individual countries over time); shading is the 95% credible interval.

**Figure 6:**
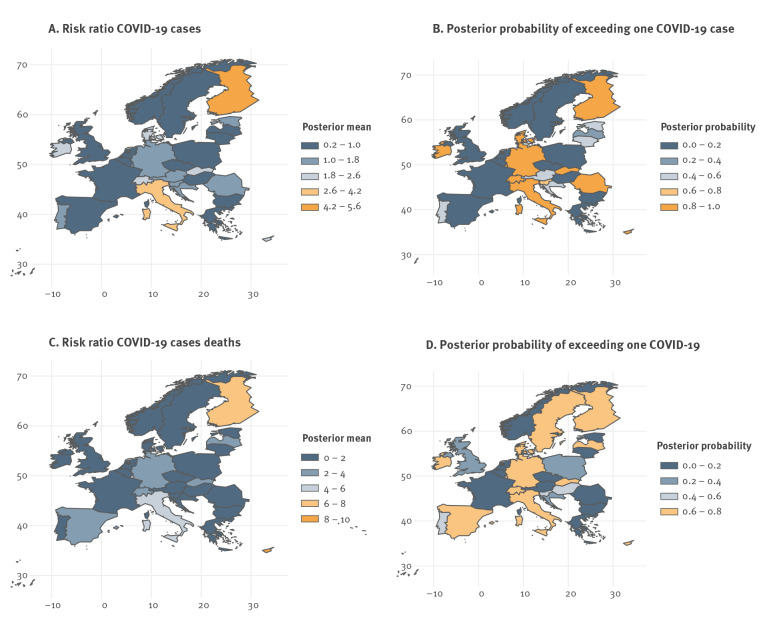
Posterior mean of the country-specific risk ratio of COVID-19 cases and deaths and posterior probability of exceeding one COVID-19 case or death, 30 European countries, 2020

Figure 6 shows the maps of the posterior mean for the country-specific relative risks of COVID-19 cases (panel A) and COVID-19 deaths (panel C). These country-specific risks enable comparison of individual countries to case/death incidence in the whole study area, having accounted for the effects of all other covariates in the model. Figures 6A and 6C indicate whether the cases or deaths per 100,000 were higher or lower in a given country relative to the incidence in the full region. Posterior means in the top two categories (shades of orange) indicate especially high excess of country-specific risk relative to cases/deaths in the whole region. Posterior means lower than 1.0 (dark blue) indicate a lower risk ratio than that of the whole region. Figures 6B and 6D show the country-specific posterior probability (range: 0–1) of observing a relative risk larger than one compared with case/death incidence in all 30 countries. The proportion of spatial variance explained by the models is 16% for the case-specific model and 15% for the death-specific model. These values (15–16%) are not high, indicating that the spatial components of the models are not highly explanatory of the variability in cases/deaths.

COVID-19: coronavirus disease.

Country-specific relative risks compared with the whole of the study area have accounted for the effects of all other covariates in the model; posterior probability of exceeding one case or death is calculated per 100,000 persons after adjusting for covariates.

### Analysis 2

For confirmation and comparison, we repeated the analysis using a multilevel mixed-effects model with results shown in Table 3. The conclusions of this analysis were broadly the same as for the hierarchical probabilistic models described above. The coefficients for these models assesses the independent contributions of the interventions to the outcomes while holding the others at their mean (as we would expect from a multivariate linear model). The incident risk ratios (IRR) are shown in Table 3 with 95% credible intervals, for either deaths or cases, for each period (each week) after the intervention started. Larger IRR values suggest greater effects; a value of 1 implies no effect, values above 1.0 suggest increase in cases/deaths, while values below 1 imply decrease. For time periods 1–7 and 8–14 days, the IRR values were above 1, indicating a positive association between cases/death and the intervention variable. For periods starting 15 days onwards the IRR was generally below 1 suggesting a negative association between the outcome and the intervention. This pattern probably reflects the time lag between exposure, latency and disease detection, so that the impact of interventions only kicks in after what is effectively a lag period of 14 days. Closing schools, banning mass gatherings and initial business closures reduced cases and deaths most. Other measures had smaller and less consistent effects. 

**Table 3:** Results of mixed-effects negative binomial model of the effect of each intervention on COVID-19 case numbers and deaths, 30 European countries, 2020

Intervention	Timing	Cases		Deaths	
		IRR	95% CI	IRR	95% CI
Mass gathering restrictions	**Before**	**1**	Reference	**1**	Reference
	1–7 days after	1.32	1.10–1.57	0.76	0.55–1.03
	8–14 days after	1.13	0.88–1.43	0.58	0.41–0.84
	15–21 days after	0.99	0.73–1.34	0.59	0.38–0.92
	22–28 days after	0.80	0.56–1.15	0.56	0.33–0.93
	29–35 days after	0.74	0.48–1.13	0.50	0.28–0.91
	≥ 36 days after	0.66	0.40–1.09	0.49	0.25–0.98
Initial business closures	**Before**	**1**	Reference	**1**	Reference
	1–7 days after	1.18	0.96–1.46	1.07	0.80–1.43
	8–14 days after	0.87	0.66–1.15	1.07	0.75–1.54
	15–21 days after	0.69	0.49–0.96	0.72	0.47–1.11
	22–28 days after	0.61	0.41–0.91	0.50	0.29–0.83
	29–35 days after	0.47	0.29–0.76	0.42	0.22–0.77
	≥ 36 days after	0.32	0.18–0.56	0.37	0.18–0.77
Educational facilities closed	**Before**	**1**	Reference	**1**	Reference
	1–7 days after	1.47	1.22–1.79	2.51	1.89–3.34
	8–14 days after	1.38	1.05–1.80	3.14	2.14–4.62
	15–21 days after	0.95	0.67–1.33	2.76	1.74–4.36
	22–28 days after	0.52	0.35–0.78	2.02	1.19–3.43
	29–35 days after	0.26	0.16–0.42	1.10	0.60–2.01
	≥ 36 days after	0.14	0.08–0.25	0.55	0.28–1.10
Non-essential services closed	**Before**	**1**	Reference	**1**	Reference
	1–7 days after	1.14	0.92–1.41	1.40	1.03–1.90
	8–14 days after	1.15	0.90–1.47	1.41	1.00–1.97
	15–21 days after	1.02	0.78–1.33	1.42	0.99–2.03
	22–28 days after	0.83	0.60–1.13	1.44	0.95–2.17
	29–35 days after	0.76	0.52–1.10	1.04	0.65–1.68
	≥ 36 days after	0.76	0.46–1.26	0.77	0.42–1.39
Stay-at-home order/advisory	**Before**	**1**	Reference	**1**	Reference
	1–7 days after	1.19	0.97–1.47	1.30	0.96–1.76
	8–14 days after	1.95	1.56–2.44	2.01	1.45–2.77
	15–21 days after	2.28	1.79–2.90	2.23	1.58–3.14
	22–28 days after	2.55	1.94–3.35	1.99	1.36–2.89
	29–35 days after	2.49	1.78–3.48	1.84	1.19–2.83
	≥ 36 days after	2.39	1.49–3.84	1.21	0.70–2.10
Mask order/advisories	**Before**	**1**	Reference	**1**	Reference
	1–7 days after	0.66	0.55–0.79	0.91	0.75–1.11
	8–14 days after	0.53	0.43–0.65	0.89	0.71–1.12
	15–21 days after	0.52	0.40–0.67	0.97	0.73–1.29
	22–28 days after	0.68	0.48–0.98	1.40	0.91–2.15
	29–35 days after	1.15	0.70–1.87	1.36	0.72–2.55
	≥ 36 days after	1.06	0.56–2.01	1.45	0.60–3.54
Days from epidemic start	Per day	1.14	1.12–1.15	1.17	1.15–1.19
Tests per 1,000 population done by 16 April 2020		1.06	1.04–1.07	1.02	0.99–0.06
Random effects					
Country (variance)		0.26	0.15–0.46	1.19	0.70–2.03

CI: confidence interval; COVID-19: coronavirus disease; IRR: incident risk ratio.

The IRR is generated by exponentiating the results of the model’s raw outputs which were generated in a default log scale.

In addition, we looked at the impact of removing one intervention at a time or all interventions on the model log likelihoods (Table 4). The biggest impact came from removing educational closures from the model. The next biggest change came from removal of stay-at-home orders, but this intervention was associated with a smaller decline in epidemic risk (deaths). We note that removing mask wearing as a control measure had a moderate effect on case counts but very minor effect in mortality outcomes; this difference may reflect the relatively late imposition of mask-wearing mandates/advisories.

**Table 4:** Log likelihood of each model for full model compared with models excluding each of the COVID-19 interventions and all interventions, 30 European countries, 2020

Model	Log likelihood	Change
Full model (cases)	−9,081	NA
Excluded		
Mass gathering restrictions	−9,096	−15
Initial business closures	−9,097	−16
Educational facilities closed	−9,157	−76
Non-essential services closed	−9,085	−4
Stay-at-home advisory	−9,112	−31
Face coverings	−9,109	−28
All interventions	−9,617	−536
Full model (deaths)	−4,096	NA
Excluded		
Mass gathering restrictions	−4,101	−5
Initial business closures	−4,109	−13
Educational facilities closed	−4,163	−66
Non-essential services closed	−4,104	−8
Stay-at-home advisory	−4,113	−17
Face coverings	−4,100	−4
All interventions	−4,569	−472

COVID-19: coronavirus disease; NA: not applicable.

### Collinearity and sensitivity analyses

Regression diagnostics for the alternative specifications of response time periods in Analysis 2 (4, 10 or 14 rather than 7 days) are shown in Supplement 2, with visual comparisons available in Supplement 1, part 6. There was little difference in the overall rate of decline in risk ratio with increased time since intervention regardless of time unit used. There were noticeable outliers in a few model IRR values at the longest time periods (more than 40 or 50 days) when data contributions tended to be from just one or two countries (see Supplement 1, part 6 and Supplement 2).

The VIF values for the predictor variables in Analysis 1 were all smaller that 10 (mean VIF 5.7) except for initial business closures which gave a VIF of 10.4 (Supplement 1, part 3). Collinearity diagnostics for Analysis 2 were almost identical, in that the VIF only just exceed the 10.0 threshold and only for the initial business closures variable (Supplement 1, part 4). The condition index exceeded 15.0 in the ninth dimension and suggested some collinearity between initial and non-essential business closure parameters. However, corresponding variance proportions in all dimensions for each control measure were well below 0.9. The smallest eigenvalue (Supplement 1, part 4) was 0.059, which is above the suggested threshold of 0.01. These tests as a group indicate that collinearity between predictor variables did not harmfully bias the apparent separate contributions of each disease control measure (as indicated by coefficient central estimates) in our models. In addition, the standard errors of the predictors in both models were small (rarely > 0.20), while in the sensitivity and collinearity checks, dropping any one of the main predictor variables from the final equation of Analysis 2 did not strongly change the coefficients and standard errors of remaining predictor variables. We conclude that there was some collinearity in our models, notably between the business closure variables, but that this was not enough to affect our conclusions.

## Discussion

Our analyses confirm that the imposition of non-pharmaceutical control measures have been effective in controlling epidemics in each investigated country. However, we were unable to demonstrate a strong impact from every intervention. Closure of educational facilities, banning mass gatherings and early closure of some but not necessarily all commercial businesses were all associated with reduction of the spread of infection. Widespread closure of all non-essential businesses and stay-at-home orders seem not to have had much additional value. Other analyses of actual intervention impositions and subsequent case/death counts have also found that school closures were especially effective control measures for reducing spread of COVID-19 [25-28]. However, it is vital that we caveat this finding (about closing educational establishments) by noting that it relates to closing schools that operated ‘as normal’ rather than when they operated with COVID-19-secure policies. We also do not attempt here to discuss what the best COVID-19 mitigation measures might be within schools.

It seems likely that many possible combinations of physical distancing measures can be effective. The apparent effects of the measures as described here may be biased by the measures themselves tending to have a sequence in common among all countries. Measures imposed later may seem less effective simply because of the order in which they happened (additional benefits were small after other measures were put in place). Other analysts have drawn this same conclusion about coronavirus NPI [28]. Our analyses indicated that school closures and stopping mass gatherings were most effective, but we acknowledge that these measures were among the earliest taken in Europe; the data did not allow us to see what marginal gains might have been achieved if school closures had been the last of all measures taken. Also, different measures reinforced and enabled each other: for instance, there was little incentive to leave home if schools and businesses were already closed and weather was inclement (as it often is in early spring in Europe, when most physical contact restrictions started). Business and school closures usually preceded stay-at-home measures in Europe, so it may not have been possible for data on stay-at-home orders to be linked to large additional effects. This potential ordering problem is at least somewhat mitigated for by our use of individual lag measures (in timing) from when each intervention was effected. It is also worth noting that outside of institutional and crowded settings, there is evidence that much, if not most, COVID-19 transmission was within households in this period [29]; stay-at-home orders intensify contact within households which would be expected to increase household transmission. It could therefore not be surprising that stay-at-home measures on their own are not very effective outbreak control measures and may not generate large additional benefits.

There has been uncertainty about how beneficial the closing of educational establishments can be on coronavirus respiratory disease transmission [28,30-35], especially given that children often have mild or no symptoms [36]. We cannot resolve the lack of consensus in these lines of evidence about how likely children are to pass severe acute respiratory syndrome coronavirus 2 (SARS-CoV-2) to adults. Emergences of novel and seemingly more infectious variants [37] of the virus may complicate attempts to understand transmission patterns from children to adults using historical data and to understand the relative effectiveness of specific non-pharmaceutical interventions. Our study similarly does not identify which level of school closure has the most benefit, whether it is primary, junior, senior school or even higher education, although more recent evidence tends to point towards schooling between the ages of 11 and 19 years as being more likely to drive transmission than education for younger children [33]. Note that our own results are based on total closure rather than schools operating with at least partial social distancing. The impacts of partial school closures or social distancing controls within open schools need to be evaluated separately.

After closing educational establishments, the next greatest impact on the epidemiology of the European COVID-19 controls was from banning mass gatherings (which could be of any size), both public and private gatherings. A 2018 review of spread of respiratory infectious disease during mass gatherings found that most evidence was linked to the Islamic Hajj pilgrimage, where infections were mainly from rhinovirus, human coronaviruses and influenza A virus [38]. The evidence for respiratory disease outbreaks arising from other mass gatherings such as music festivals or sporting events is less established, but not absent. Several outbreaks of respiratory infectious disease have been linked to large festivals [38,39]. For instance, during the 2009/10 influenza season, pandemic influenza A(H1N1)pdm09 outbreaks were recorded at three of Europe’s six largest music festivals, while some 40% of pandemic influenza cases that season in Serbia were linked to the Exit music festival. Analysis of COVID-19 NPI by other investigators using different approaches than ours also tend to find that banning large gatherings can be especially effective for reducing disease transmission [28].

The types of business closures are interesting. We established that there was weak collinearity between the two types of business closures in the models. However, the stronger association between a business closure control measure and case declines was with the initial business closures. Given that those initial closures were mostly directed at business where people congregate and that have a purpose of facilitating socialising (i.e. the hospitality industry), this would suggest that control measures among these businesses are where the most impact may be had. Although outbreaks of food poisoning are frequently linked with venues where food is consumed, this is much less frequent for outbreaks of respiratory infections. One exception was an outbreak of SARS at a restaurant where live palm civets were caged close to customer seating [40]. The link with COVID-19 is probably less about food and beverage consumption than about time people spend in close proximity to each other.

Similar to some other authors who have tried to assess relative importance of possible NPI in controlling COVID-19 and not found strong benefits for face-cover usage [41], we hesitate to interpret our findings on mask wearing as definitive. Mask advisories have not been implemented in isolation and were often implemented relatively late in the sequence of NPI in the group of European countries that we studied. Mask interventions were also implemented unevenly (as advisories or mandates) and usually only in limited settings. Our separate evidence review [42] found that mask wearing to stop respiratory disease transmission is likely to be only modestly effective, but we agree that when it comes to a pandemic situation, small protective measures may have cumulative important benefits [43].

Our study had limitations. Although our results suggests that closures of educational interventions and banning mass gatherings are the most important measures, this is caveated with several observations. Many interventions were implemented in different ways and at different points in the local epidemic. We relied on published and observed data which may have suffered from problems of under-ascertainment; the true effect of specific interventions may depend on true community prevalence that was not measured accurately enough. We did not undertake a systematic sensitivity analysis (excluding just one country per model, for instance) or adjustments in categorisations. It is likely that there will be serial dependency in the data as the level of disease at one time point is (inevitably) dependent on prior states of disease in the country, but we did not attempt to measure serial dependency in our models which might have further informed relative NPI efficacy. For example, in accordance with the IHME assignment, we treated Sweden as a country without school closures because schools for persons under 16 stayed open, although upper secondary and tertiary education facilities were actually shut in Sweden from late March 2020 [34]. Given recent evidence that secondary (age 11–19 years) rather than junior schools may play an important role in transmission of COVID-19, the educational closures in Sweden may explain in part the divergence from our predictions in that country [33]. Our models cannot allow for differences between countries regarding construction materials or ventilation rates in school buildings, which might influence transmissibility. The findings in support of school closures to contain the virus can truly only refer to schools when schools operate ‘as normal’ and not with COVID-19 mitigation practices in place. The exact timing of restrictions as reported by IHME being introduced varied over time in Italy, Spain and between individual federal states in Germany. Which types of work places could stay open varied; the acceptable reasons for being outdoors also varied between countries. Stay-at-home orders in some countries were an advisory but not enforced while elsewhere they were enforced by police with penalties. In some countries, children could go outside and outdoor exercise was permitted, while in others either or both might be banned. In some countries, severe travel restrictions were a separate intervention, while in others travel bans were a consequence of a stay-at-home order and could not be identified separately. Because of this variety in how interventions were implemented and described, the results for the potential of stay-at-home advisories in particular may be underestimated. All models are simplifications of the complex nature of reality; our modelling was unable capture many subtle variations in how control measures were implemented. We acknowledge that lack of direct observation of these variations may have biased our results.

## Conclusion

Relaxing stay-at-home orders and allowing reopening of non-essential businesses appeared to be the lowest risk measures to relax as part of plans to carefully lift COVID-19 lockdown measures. The pandemic started with little clear empirical evidence on the relative value of different interventions. Yet the reasons to implement only minimal control measures were compelling, given the social and economic harm linked to tight control measures. While we need to be cautious about using preliminary results, public health officials will have to use evidence as it emerges rather than wait for a final full view to decide what might be (was) the best control strategy. Careful monitoring of how relaxation of each control measure affects transmissibility of COVID-19 is required and will help to minimise the inevitably imperfect results. 

## Supplementary Data

1052000 Supplement1

## Supplementary Data

1052000 Supplement2
