# Supplementary material for: Impact of non-pharmaceutical interventions against COVID-19 in Europe in 2020: a quasi-experimental non-equivalent group and time series design study
Source: Euro Surveill. 2021 Jul 15;26(28):2001401. doi: 10.2807/1560-7917.ES.2021.26.28.2001401 (PMC8284045; doi:10.2807/1560-7917.ES.2021.26.28.2001401)

Impact of non-pharmaceutical interventions against COVID-19

This supplementary material is hosted by *Eurosurveillance* as supporting information alongside the article "Impact of non-pharmaceutical interventions against COVID-19 in Europe: a quasi-experimental non-equivalent group and time-series design study ", on behalf of the authors, who remain responsible for the accuracy and appropriateness of the content. The same standards for ethics, copyright, attributions and permissions as for the article apply. Supplements are not edited by *Eurosurveillance* and the journal is not responsible for the maintenance of any links or email addresses provided therein.

SUPPLEMENTARY MATERIAL part 1. Dates that restrictions were imposed in states/regions of Italy, Spain, and Germany

Dates in March when restrictions were imposed: eg., 15 = 15 March,

Spain	massgath	initbuscl	educclosed	nonessent	SAHO
Andalucia	15	15	14	15	15
Aragon	15	15	14	15	15
Asturias	15	15	14	15	15
Islas B	15	15	14	15	15
Basque C	15	15	14	15	15
Canaries	15	15	14	15	15
Cantabria	15	15	14	15	15
C&Leon	15	15	14	15	15
C-LaM	15	15	14	15	15
Catalonia	11	12	14	15	15
Ceuta	15	15	14	15	15
C Madrid	15	13	11	13	15
Extremadura	15	15	14	15	15
Galicia	15	15	14	15	15
La Rioja	15	15	14	15	15
Melilla	15	15	14	15	15
Murcia	15	15	14	15	15
Navarre	15	15	14	15	15
C Valencian	15	15	14	15	15
medians	15	15	14	15	15

SUPPLEMENTARY MATERIAL part 1. Dates that restrictions were imposed in states/regions of Italy, Spain, and Germany (continued)

Dates in March when restrictions were imposed: eg., 11 = 11 March, -7 = 7 days before 1 March, or 23 Feb.

Italy	massgath	initbuscl	educclosed	nonessent	SAHO
Abruzzo	11	11	5	11	11
Basilicata	11	11	5	11	11
Calabria	11	11	5	11	11
Campania	11	11	5	11	11
Emilia-R	7	7	1	11	11
F-V Giulia	11	11	5	11	11
Lazio	11	11	5	11	11
Liguria	11	11	5	11	11
Lombardia	-7	-7	1	8	8
Marche	7	7	5	11	11
Molise	11	11	5	11	11
Piemonte	7	7	5	11	11
di Bolzano	11	11	5	11	11
di Trento	11	11	5	11	11
Puglia	11	11	5	11	11
Sardegna	11	11	5	11	11
Sicilia	11	11	5	11	11
Toscana	11	11	5	11	11
Umbria	11	11	5	11	11
V d'Aosta	11	11	5	11	11
Veneto	-7	-7	1	11	11
medians	11	11	5	11	11

SUPPLEMENTARY MATERIAL part 1. Dates that restrictions were imposed in states/regions of Italy, Spain, and Germany (continued)

Dates in March when restrictions were imposed: eg., 22 = 22 March, -3 = 3 days before 1 March, or 27 Feb.

	massgath	initbuscl	educclosed	nonessent	SAHO
Germany	21	21	17	21	21
	21	17	16	21	21
	23	14	23	23	23
	23	17	18	17	17
	17	20	16	20	22
	22	15	16		22
	22	15	16		22
	23	27	16	27	23
	23	18	16	18	23
	23	-3	16	23	23
	22	23	16	23	22
	21	15	16		21
	22	24	16	24	22
	23	23	23	23	23
	24	14	16	24	24
	22	15	17		22
medians	22	17	16	23	22

SUPPLEMENTARY MATERIAL part 2: Orders or recommendations to wear facemasks or face coverings, by sovereign territory in Europe

Austria 6 April 2020 (compulsory)

Compulsory in shops and most commercial premises from 6 April 2020, soon widened to public transport and shops that were due to re-open on 14 April.

https://uk.reuters.com/article/us-health-coronavirus-austria/austria-to-make-basic-face-masks-compulsory-in-supermarkets-idUKKBN21H16A

https://uk.reuters.com/article/uk-health-coronavirus-austria-masks/austrian-supermarkets-hand-out-face-masks-before-they-become-compulsory-idUKKBN21J5XP

=====================

Bulgaria 30 March 2020 (mix recommended/compulsory)

Strong recommendation from 30 March; made compulsory in all public places from 12 April; extended to 13 May.

https://sofiaglobe.com/2020/03/30/covid-19-bulgaria-makes-wearing-a-protective-mask-in-public-places-compulsory/

https://www.bnr.bg/en/post/101257255/bulgaria-introduces-mandatory-wearing-of-masks-in-public-from-april-12-until-april-26-inclusive

https://www.novinite.com/articles/204264/The+Mandatory+Wearing+of+Protective+Masks+is+Extended+until+May+13

===========================

Czechia 18 March 2020 (compulsory)
Mandatory in all public spaces and many work places. Order extended until end of June 2020.

https://www.praguemorning.cz/face-masks-now-mandatory-in-all-prague-shops-and-offices/

https://news.expats.cz/weekly-czech-news/prymula-face-masks-to-remain-mandatory-in-czech-republic-until-end-of-june/

====================
Estonia 5 April 2020 (highly recommended) statement by PM

https://news.err.ee/1073236/prime-minister-we-are-unfortunately-still-in-coronavirus-deepening-phase

========================

France 5 April (encouraged not required)

https://www.thelocal.fr/20200406/mask-or-no-mask-what-is-the-official-coronavirus-advice-in-france

https://www.france24.com/en/20200405-coronavirus-abrupt-reversal-on-mask-policy-in-france-and-the-us-raises-new-questions
=======================

Germany 1 April 2020 (recommendation that became compulsory)

Variable rules for when must be worn vary by state and sometimes by city, introduced dates also variable. Nationally mandated from 27 April on public transport & also in most shops before then. RKI formally endorsed mask-wearing on 1 April.

https://www.thelocal.de/20200402/latest-face-masks-in-public-could-help-to-reduce-spread-of-coronavirus-says-germanys-robert-koch-institute

https://muscateer.om/en/news/europe-updategermany-new-face-mask-rules-in-idZ2trbg==

=======================

Italy 6 April mandatory in some regions, some settings by 6 April, endorsed by national govt previously but uneven uptake.

Lombardy, Tuscany 6 April mandatory anywhere outdoors

https://www.ansa.it/english/news/2020/04/06/coronavirus-lombardy-makes-face-masks-compulsory_a852ffdb-a0dd-4c55-a725-e852c5a2fc43.html

https://uk.reuters.com/article/us-health-coronavirus-italy-masks/scramble-for-masks-as-italian-region-orders-coronavirus-cover-up-idUKKBN21O1Y0

https://www.thelocal.it/20200406/coronavirus-where-should-you-wear-a-face-mask-in-italy

========================
Lithuania 1 April (recommendation)

https://www.ecdc.europa.eu/sites/default/files/documents/COVID-19-use-face-masks-community.pdf

====================

Luxembourg 20 April 2020 (**compulsory in some situations**, where can’t keep 2m apart)

https://uk.reuters.com/article/us-health-coronavirus-luxembourg/luxembourg-enforces-use-of-masks-as-lockdown-eases-idUKKBN2221W3

=====================
Norway 5 April (encouraged)

https://www.newsinenglish.no/2020/04/05/officials-change-their-minds-about-masks/

https://www.fhi.no/en/op/novel-coronavirus-facts-advice/facts-and-general-advice/hand-hygiene-cough-etiquette-face-masks-cleaning-and-laundry/

=====================
Poland 16 April (mandatory, most public places)
https://www.aljazeera.com/news/2020/04/countries-wearing-face-masks-compulsory-200423094510867.html

=====================
Slovakia 14 March (recommendation, followed by requirement from about 1 April)

https://balkaninsight.com/2020/04/09/slovak-news-crews-hailed-for-covid-19-coverage/

https://www.npr.org/sections/coronavirus-live-updates/2020/04/01/825180019/in-big-adjustment-some-european-countries-push-for-residents-to-wear-masks

https://balkaninsight.com/2020/04/09/slovak-news-crews-hailed-for-covid-19-coverage/

https://www.ecdc.europa.eu/sites/default/files/documents/COVID-19-use-face-masks-community.pdf

=========================
Slovenia 29 March (**mandatory** in many places)

https://english.sta.si/2746850/slovenia-sticking-to-use-of-masks-in-indoor-public-places

=========================

Spain 13 April (**recommended** and sometimes freely given out, ie train stations, but not compulsory)

https://www.rtve.es/noticias/20200413/como-colocar-retirar-desechar-mascarillas-higienicas-para-evitar-contagio-coronavirus/2011879.shtml

https://www.thelocal.es/20200424/what-are-the-rules-for-wearing-a-protective-mask-in-spain

===================================

Countries with no predominant government recommendation or compulsion in place (as of noon 29.4)
Belgium, Croatia, Republic of Cyprus (south), Denmark, Finland, Greece, Hungary, Ireland, Latvia, Malta, Netherlands, Portugal (likely soon), Romania, Sweden, Switzerland, UK

Netherlands Masks are not a substitute for 1.5 m
https://www.dutchnews.nl/news/2020/04/dutch-stay-firm-on-face-masks-but-they-may-have-an-exit-strategy-role/

Hungary 27 April mandatory, commuters & shoppers but in Budapest only
https://www.themayor.eu/en/budapest-makes-masks-mandatory-for-shoppers-and-commuters

UK
Scotland's first minister recommended (did not mandate) that face coverings (not surgical grade masks) should be worn in all enclosed public spaces, from 28 April 2020. Because Scotland comprises 5.5 million (just 8.2% of the total UK population of approximately 67.9 million) we still (as of 29.4.20) treated the entirety of the UK as a country without an official endorsement of face coverings in our modelling.

https://www.gov.scot/publications/coronavirus-covid-19-public-use-of-face-coverings/

SUPPLEMENTARY MATERIAL part 3: Variance Inflation Factors (VIF) for Analysis 1, run as linear models

Variance inflation factors	
mass_gathering_restrictions	4.915550
initial_business_closure	10.011556
education_facilities	9.490315
non_essential_services	6.253501
stay_home	3.514925
masks	1.432351
tests per million population as of 16 April 2020	1.295814

SUPPLEMENTARY MATERIAL part 4: Collinearity Diagnostics

Variance inflation factors for Analysis 2 model fit as linear regression

Variable	VIF	1/VIF
Initial business closures	10.44	0.095815
Education closures	9.87	0.101302
Mass gatherings banned	6.52	0.153397
Non essential business closures	6.27	0.159552
Days elapsed into main epidemic	6.15	0.162476
Stay at home order	3.52	0.284366
Mask advisory/mandate imposed	1.47	0.680742
Tests per 1 million/population by 16 April	1.31	0.764803
Mean VIF	5.69	

SUPPLEMENTARY MATERIAL part 4: Collinearity Diagnostics (continued)
Analysis 2 Model when fit as linear regression, yielded condition indices and variance-decomposition proportions

**Dimension ->**	**1**	**2**	**3**	**4**	**5**	**6**	**7**	**8**	**9**
Condition indices ->	**1**	**2.61**	**3.56**	**5.08**	**6.04**	**9.37**	**11.44**	**14.71**	**15.9**
Model Parameter					**Variance proportions**				
Constant	0	0.03	0.01	0.01	0.31	0.21	0.14	0.26	0.03
Mass gathering restriction	0	0	0	0.06	0	0.02	0.49	0.13	0.3
Initial business closure	0	0	0	0	0.03	0.01	0.27	0.04	**0.65**
Education facilities closed	0	0	0	0.01	0.02	0.04	0.1	0.33	0.5
Non essential services closed	0	0	0	0.05	0.02	0.61	0.15	0.01	**0.15**
Stay at home order	0	0.02	0.04	0.2	0.23	0.48	0	0.01	0.03
Masks advisory or mandated	0	0.17	0.72	0.01	0.02	0.04	0	0.02	0.01
Days elapsed from start main epidemic	0	0	0	0.01	0	0	0	0.78	0.2
Tests/1 mln population to 16 April	0	0.09	0.07	0.2	0.15	0.26	0.19	0	0.03

SUPPLEMENTARY MATERIAL part 4: Collinearity Diagnostics (continued)

Principal components/correlation

Number of observations = 1588

Component	Eigenvalue	Difference	Proportion	Cumulative
Comp1	5.2667	4.21318	0.6583	0.6583
Comp2	1.05353	0.302265	0.1317	0.79
Comp3	0.751261	0.26581	0.0939	0.8839
Comp4	0.485451	0.3267	0.0607	0.9446
Comp5	0.158751	0.041833	0.0198	0.9645
Comp6	0.116918	0.008604	0.0146	0.9791
Comp7	0.108315	0.049241	0.0135	0.9926
Comp8	0.059073	.	0.0074	1

SUPPLEMENTARY MATERIAL part 5: Please see separate document for full regression model specifications and outputs. **Alternative results using different time step units for epidemic response, Analysis 2.** Tested variations are 4 days, 7 days, 10 days or 14 day units. The main manuscript describes the results when using 7 day time response periods, as there was little difference in the overall trends of a decline in risk ratios with time since interventions were imposed. Please see Supplementary Material 6 (below) for further detail showing between model comparisons.

SUPPLEMENTARY MATERIAL part 6: Alternative results using different time step units for epidemic response, Analysis 2: Comparisons of Incident Risk Ratios (IRRs) when models were generated using different time step response units: 4, 7, 10 or 14 days.

6A: IRRS for CASES

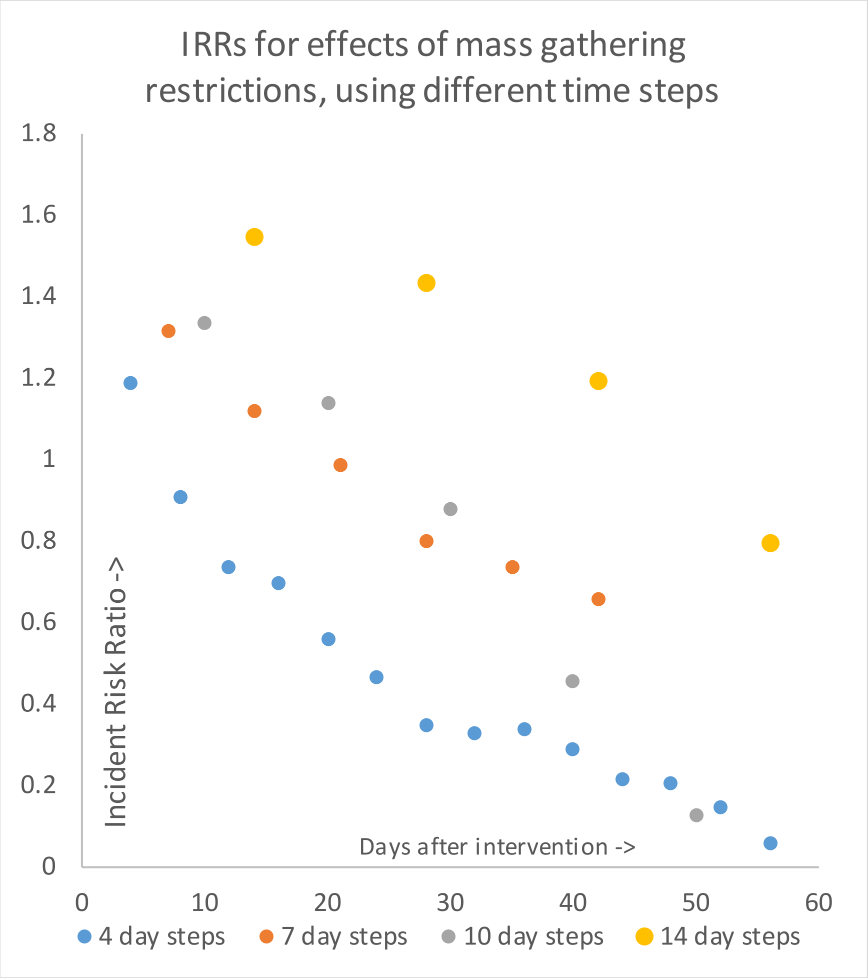

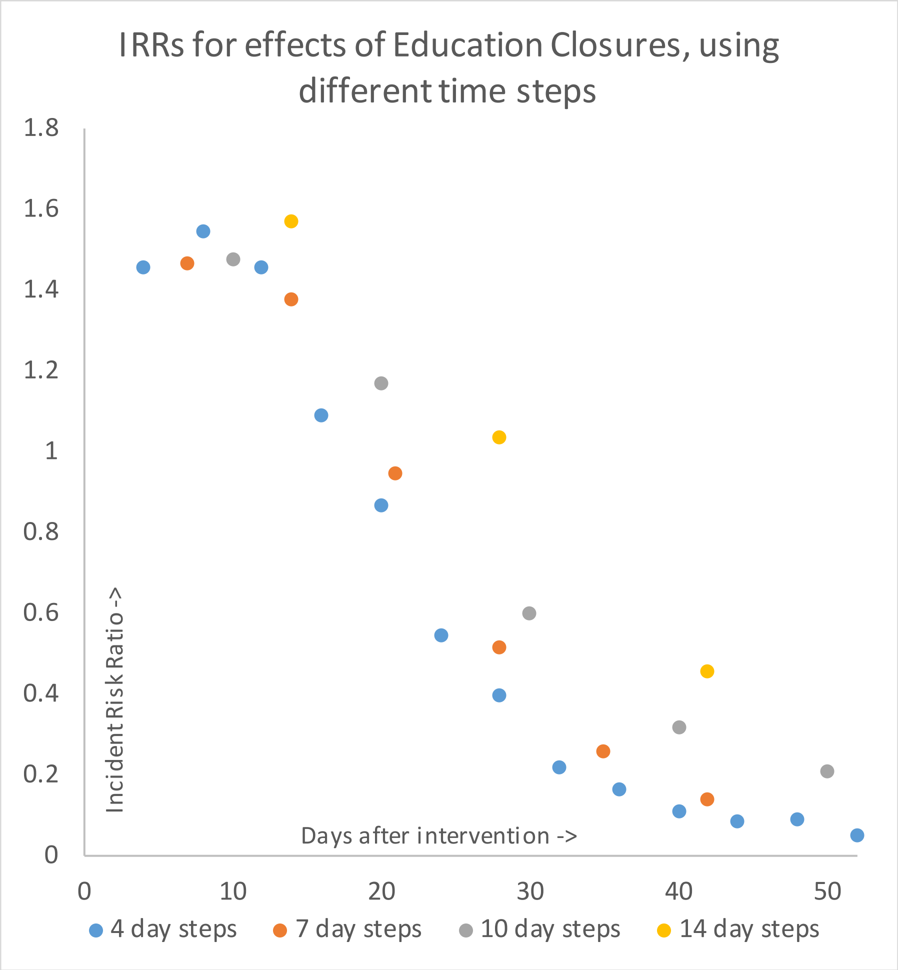

6A: IRRS for CASES (continued)

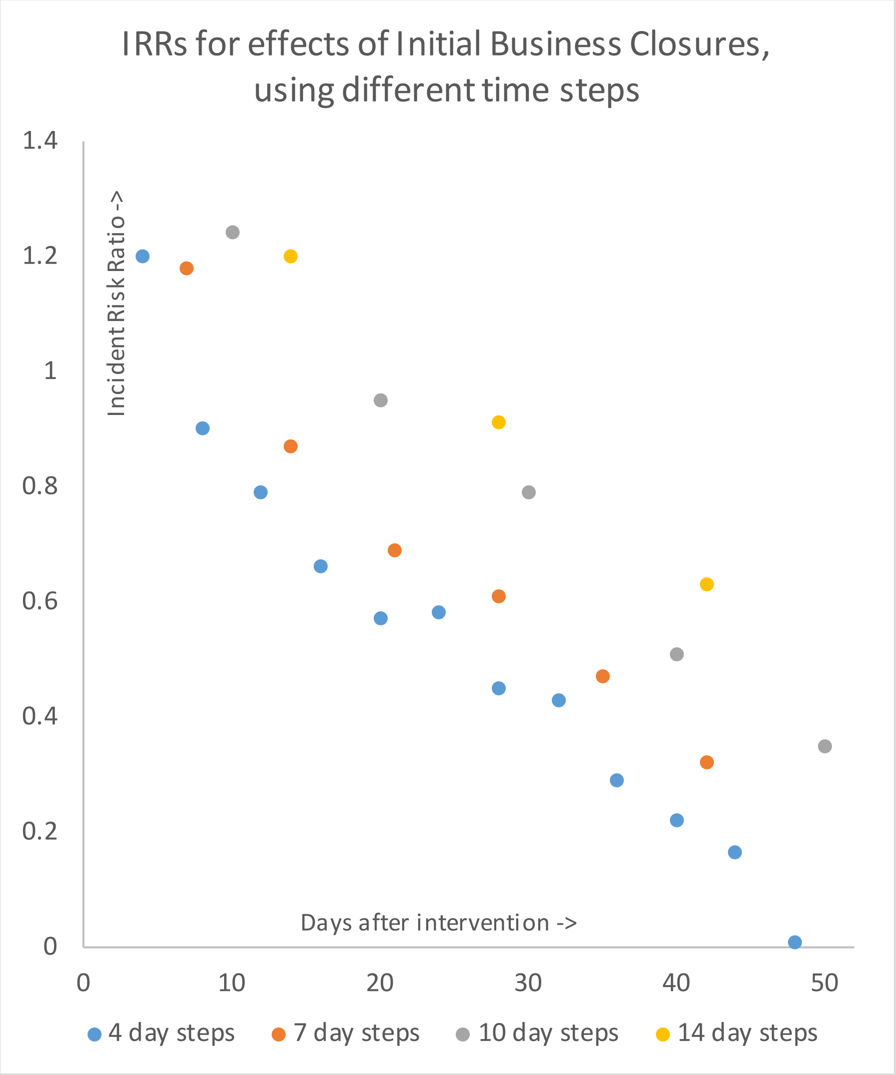

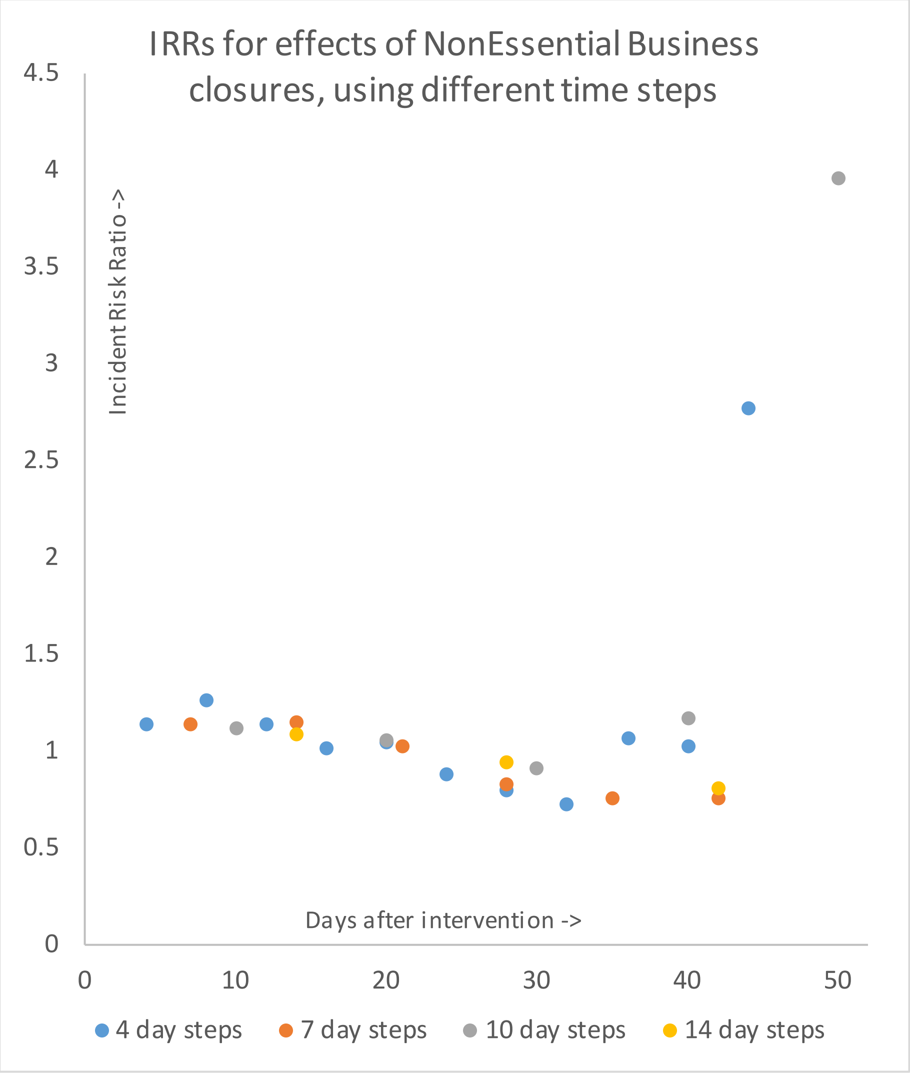

6A: IRRS for CASES (continued)

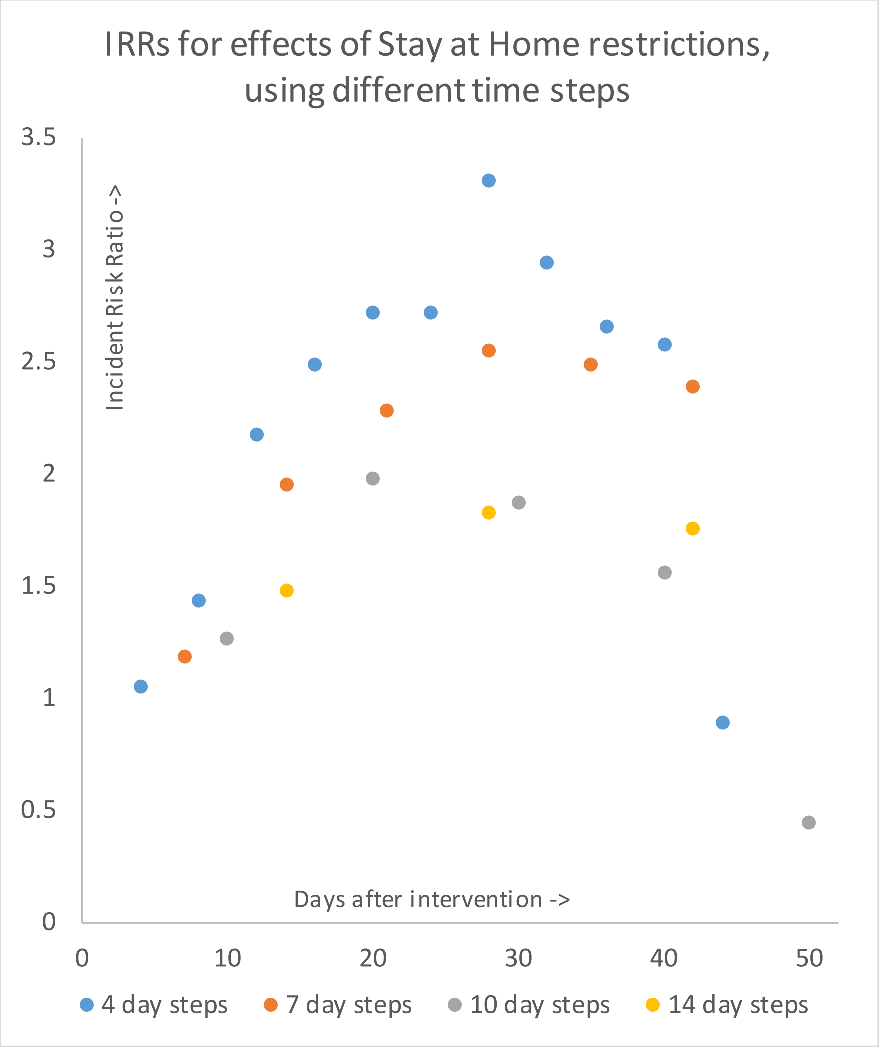

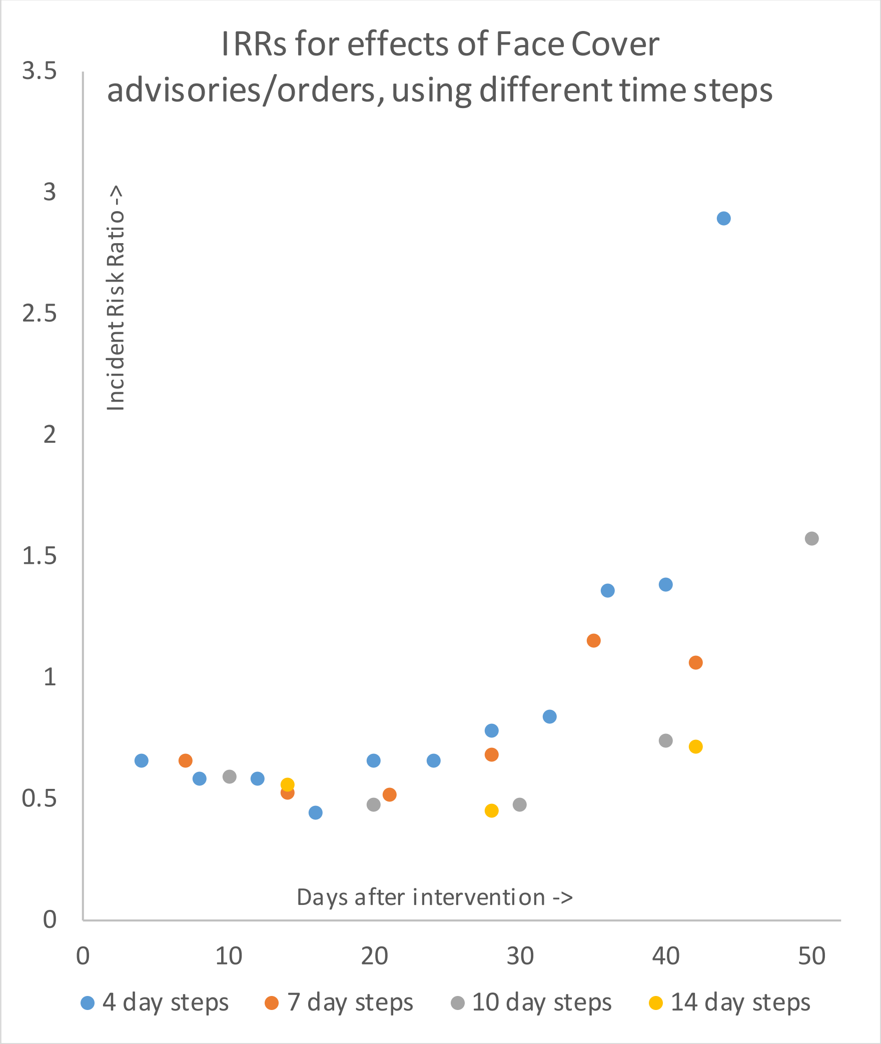

6B: IRRS for DEATHS

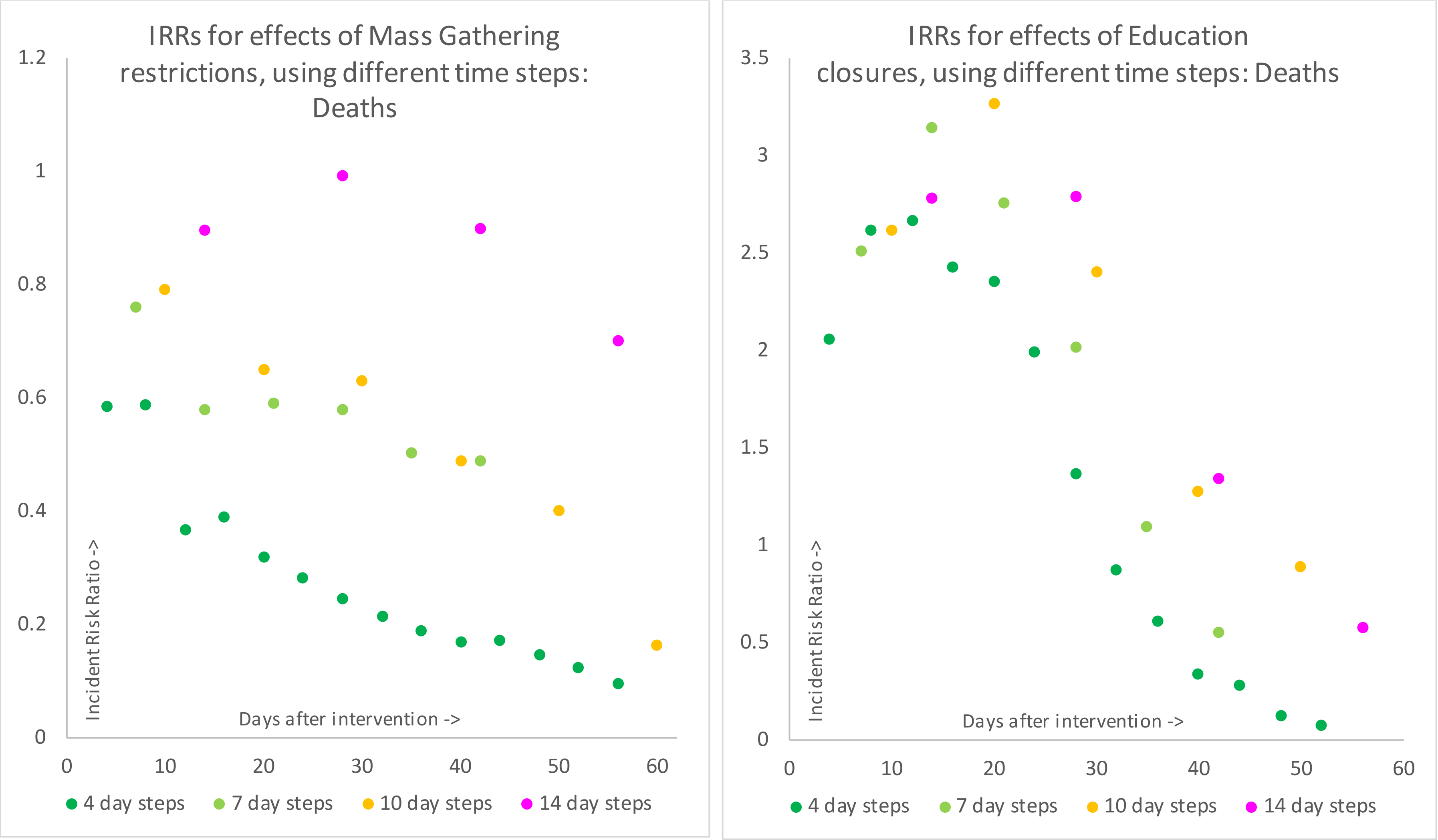

6B: IRRS for DEATHS (continued)

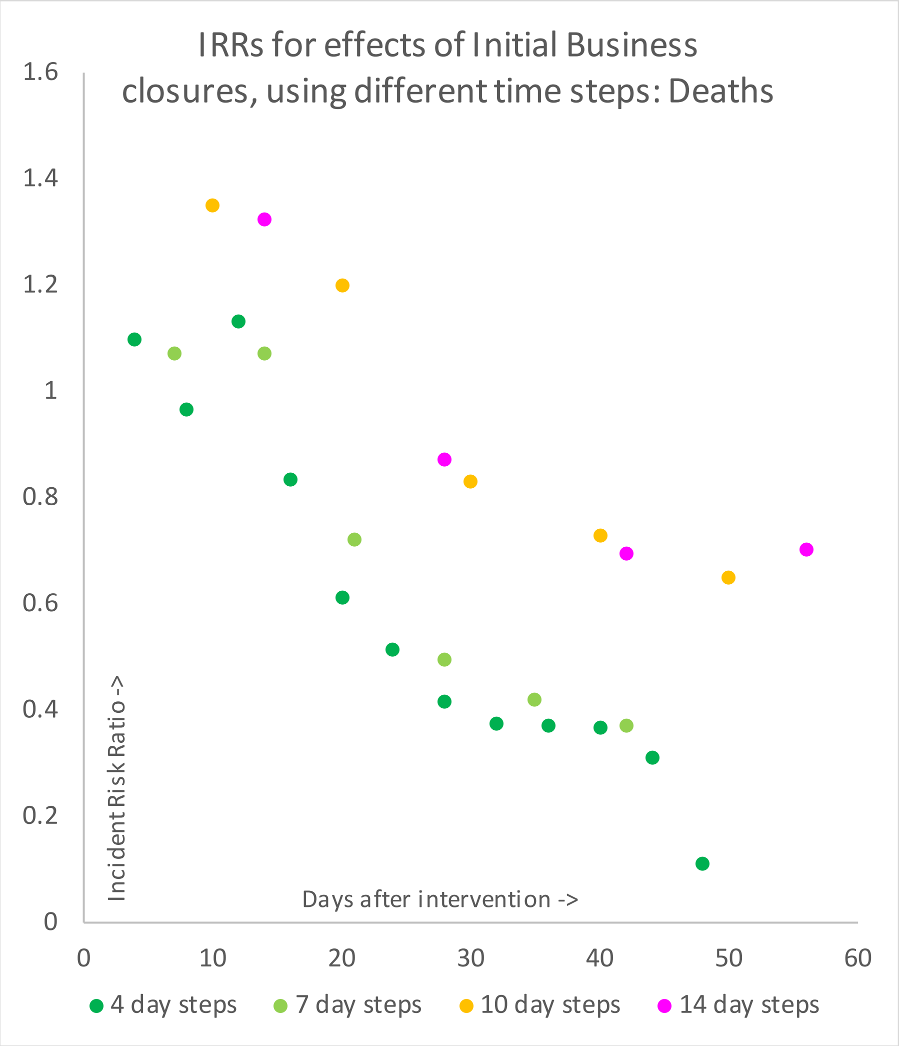

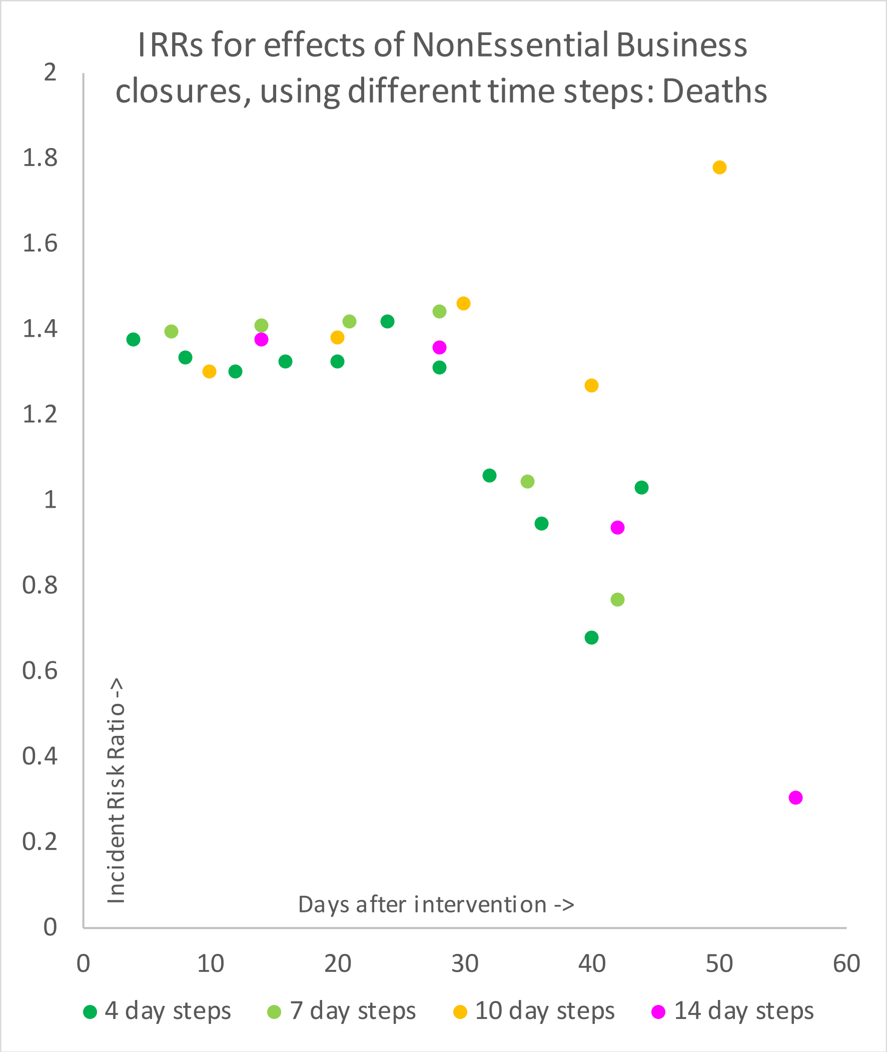

6B: IRRS for DEATHS (continued)

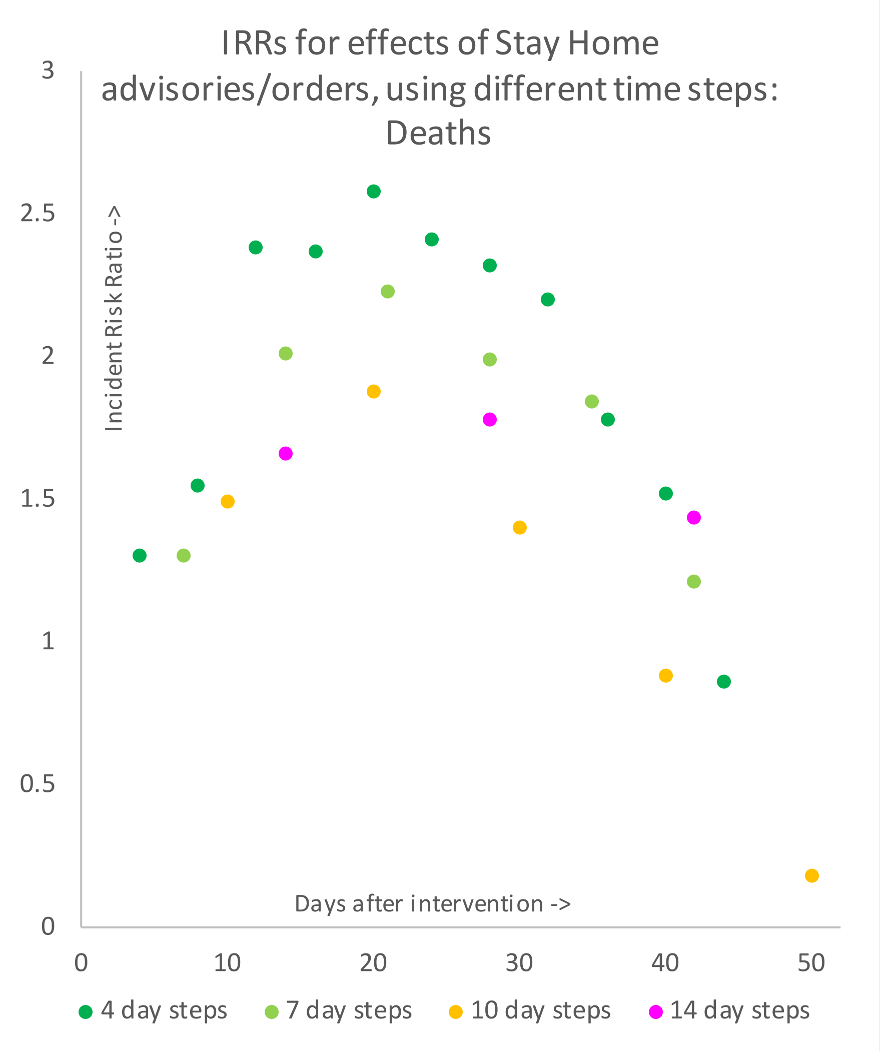

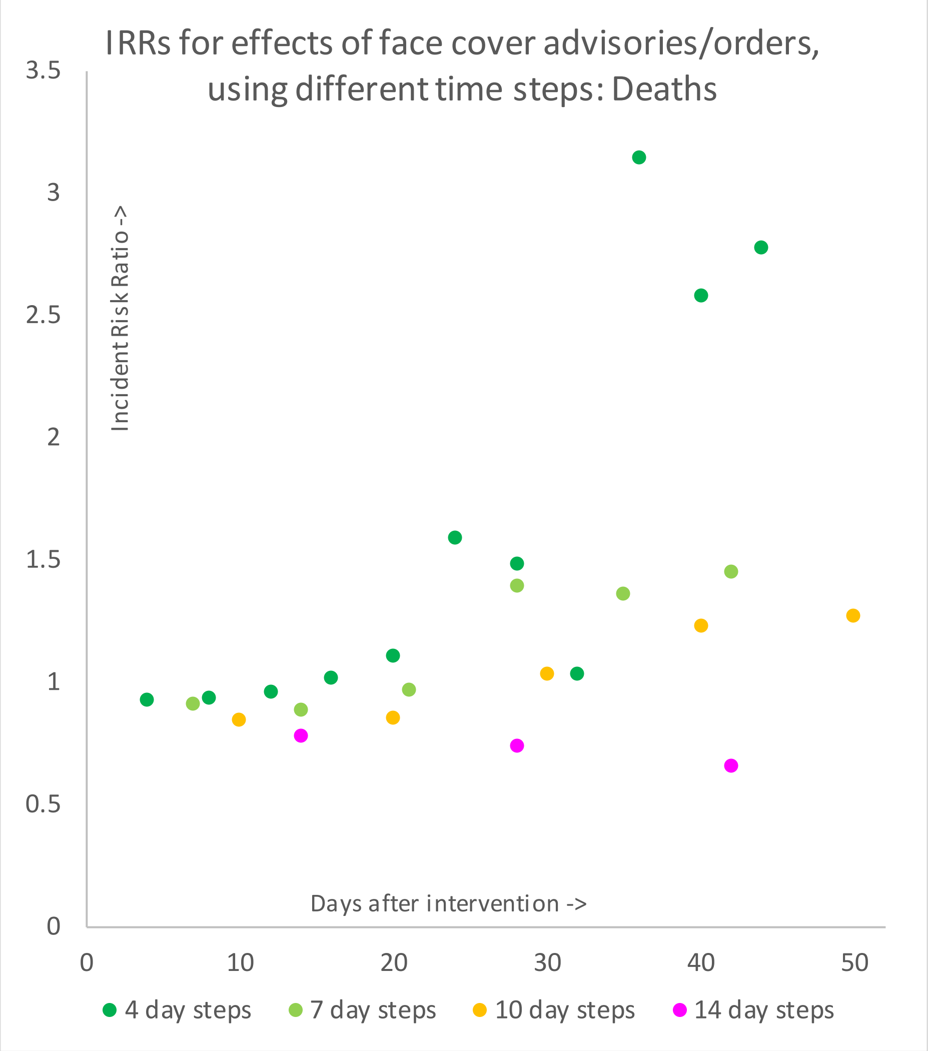

Supplement: Supplement1 [file 20-01401_BRAINARD_Supplement1.pdf]
