# Supplementary material for: Impact of non-pharmaceutical interventions against COVID-19 in Europe in 2020: a quasi-experimental non-equivalent group and time series design study
Source: Euro Surveill. 2021 Jul 15;26(28):2001401. doi: 10.2807/1560-7917.ES.2021.26.28.2001401 (PMC8284045; doi:10.2807/1560-7917.ES.2021.26.28.2001401)
Supplement: Supplement2 [file 20-01401_BRAINARD_Supplement2.pdf]

Impact of non-pharmaceutical interventions against COVID-19

This supplementary material is hosted by *Eurosurveillance* as supporting information alongside the article "Impact of non-pharmaceutical interventions against COVID-19 in Europe: a quasi-experimental non-equivalent group and time-series design study ", on behalf of the authors, who remain responsible for the accuracy and appropriateness of the content. The same standards for ethics, copyright, attributions and permissions as for the article apply. Supplements are not edited by *Eurosurveillance* and the journal is not responsible for the maintenance of any links or email addresses provided therein.

```
                                                      ___  ____  ____  ____  ____(R)
                                                     /__    /   ____/   /   ____/
                                                    ___/   /   /___/   /   /___/
                                                      Statistics/Data analysis

Iteration 7:   log likelihood = -9080.8363
      name:  <unnamed>
       log:  C:\Users\PaulH\Dropbox\001Covid19 files\Sensitivity analyses with varying time gaps.smcl
  log type:  smcl
 opened on:  28 Feb 2021, 18:00:34

1 . menbreg cases ib0.mass ib0.business ib0.education ib0.non_essential ib0.home ib0.masks dayfromfirst tests1mpop16ap

Fitting fixed-effects model:

Iteration 0:   log likelihood =  -9994.792  (not concave)
Iteration 1:   log likelihood = -9728.5026
Iteration 2:   log likelihood = -9423.4215
Iteration 3:   log likelihood = -9289.1035
Iteration 4:   log likelihood = -9287.8567
Iteration 5:   log likelihood =  -9287.854
Iteration 6:   log likelihood =  -9287.854

Refining starting values:

Grid node 0:   log likelihood = -9150.7276

Fitting full model:

Iteration 0:   log likelihood = -9150.7276  (not concave)
Iteration 1:   log likelihood = -9144.1122  (not concave)
Iteration 2:   log likelihood = -9137.8038
Iteration 3:   log likelihood = -9107.7886
Iteration 4:   log likelihood = -9081.8283
Iteration 5:   log likelihood =  -9080.841
Iteration 6:   log likelihood = -9080.8363
Iteration 7:   log likelihood = -9080.8363

Mixed-effects nbinomial regression             Number of obs     =      1,588
Overdispersion:            mean
Group variable:     country_code               Number of groups  =         30

                                               Obs per group:
                                                             min =         42
                                                             avg =       52.9
                                                             max =         63

Integration method: mvaghermite                Integration pts.  =          7

                                               Wald chi2(38)     =    3579.53
Log likelihood = -9080.8363                    Prob > chi2       =     0.0000
```

cases	IRR	Std. Err.	z	P>|z|	[95% Conf.	Interval]
mass						
1	1.317029	.1196723	3.03	0.002	1.102175	1.573765
2	1.125023	.1379665	0.96	0.337	.8846566	1.430698
3	.9909768	.1532647	-0.06	0.953	.7318406	1.34187
4	.7990745	.1476335	-1.21	0.225	.5563179	1.147761
5	.7410227	.1605023	-1.38	0.166	.484691	1.132917
6	.6604644	.1673839	-1.64	0.102	.4019077	1.085357
business						
1	1.183943	.1265193	1.58	0.114	.9602165	1.459797
2	.8705053	.1228561	-0.98	0.326	.6601456	1.147898
3	.689605	.1169872	-2.19	0.028	.4945376	.9616155
4	.6074017	.1245851	-2.43	0.015	.4063355	.907961
5	.4714363	.1140889	-3.11	0.002	.2933795	.7575586
6	.3212351	.0919537	-3.97	0.000	.1833016	.562963
education						
1	1.47373	.1447278	3.95	0.000	1.215698	1.786529
2	1.376326	.1895708	2.32	0.020	1.050702	1.802865
3	.9470467	.1642194	-0.31	0.754	.6741735	1.330366
4	.5207754	.1083542	-3.14	0.002	.3463752	.7829864
5	.2582716	.062911	-5.56	0.000	.1602275	.4163096
6	.1433753	.0402787	-6.91	0.000	.0826694	.2486591

```
non_essent~l |
          1  |   1.142639   .1234961     1.23   0.217     .9245092    1.412234
          2  |   1.146635   .1439661     1.09   0.276     .8965042    1.466554
          3  |   1.018911   .1389382     0.14   0.891     .7799502    1.331085
          4  |   .8253778   .1312559    -1.21   0.228     .6043524    1.127237
          5  |    .759917   .1443942    -1.44   0.148     .5236327    1.102822
          6  |   .7628685   .1966775    -1.05   0.294     .4602552    1.264447
             |
        home |
          1  |   1.194981   .1243872     1.71   0.087     .9744476    1.465426
          2  |   1.951905    .224044     5.83   0.000     1.558676    2.444339
          3  |   2.275837   .2798113     6.69   0.000     1.788492    2.895979
          4  |   2.550771   .3529303     6.77   0.000     1.944901    3.345381
          5  |   2.489913   .4261743     5.33   0.000     1.780296     3.48238
          6  |   2.393275   .5782635     3.61   0.000     1.490476    3.842908
             |
       masks |
          1  |   .6583008   .0615543    -4.47   0.000     .5480662    .7907074
          2  |    .528956   .0566382    -5.95   0.000     .4288218    .6524726
          3  |   .5206164   .0675501    -5.03   0.000     .4037144    .6713693
          4  |   .6819353   .1249588    -2.09   0.037     .4761763     .976604
          5  |   1.146066   .2871408     0.54   0.586     .7013678    1.872724
          6  |   1.059821   .3458243     0.18   0.859     .5590897    2.009016
             |
 dayfromfirst|   1.138656   .0074957    19.72   0.000     1.124059    1.153442
 tests1mpop~r|   1.000055   8.54e-06     6.46   0.000     1.000038    1.000072
        _cons|   2.93e-07   5.09e-08   -86.48   0.000     2.08e-07    4.12e-07
ln(pop~2018) |          1  (exposure)
-------------+----------------------------------------------------------------
    /lnalpha |  -.7180626   .0377877                     -.7921251       -.644
-------------+----------------------------------------------------------------
country_code |
   var(_cons)|   .2640212   .0740418                      .1523803    .4574554
------------------------------------------------------------------------------
Note: Estimates are transformed only in the first equation.
Note: _cons estimates baseline incidence rate (conditional on zero random effects).
LR test vs. nbinomial model: chibar2(01) = 414.04     Prob >= chibar2 = 0.0000

2 . menbreg cases ib0.mass4 ib0.business4 ib0.education4 ib0.non_essential4 ib0.home4 ib0.masks4 dayfromfirst tests1mp

Fitting fixed-effects model:

Iteration 0:   log likelihood = -9986.8369  (not concave)
Iteration 1:   log likelihood =  -9718.087
Iteration 2:   log likelihood = -9455.4816
Iteration 3:   log likelihood = -9260.6121
Iteration 4:   log likelihood =  -9258.192
Iteration 5:   log likelihood =  -9258.184
Iteration 6:   log likelihood =  -9258.184

Refining starting values:

Grid node 0:   log likelihood =  -9117.515

Fitting full model:

Iteration 0:   log likelihood =  -9117.515  (not concave)
Iteration 1:   log likelihood = -9110.9696  (not concave)
Iteration 2:   log likelihood = -9104.6382
Iteration 3:   log likelihood = -9059.2946
Iteration 4:   log likelihood = -9020.1352
Iteration 5:   log likelihood = -9015.6862
Iteration 6:   log likelihood = -9014.9553
Iteration 7:   log likelihood = -9014.9141
Iteration 8:   log likelihood =  -9014.914

Mixed-effects nbinomial regression              Number of obs     =      1,588
Overdispersion:            mean
Group variable:    country_code                 Number of groups  =         30
```

```
                                                Obs per group:
                                                              min =         42
                                                              avg =       52.9
                                                              max =         63

Integration method: mvaghermite                 Integration pts.  =          7

                                                Wald chi2(74)     =    4123.30
Log likelihood =  -9014.914                     Prob > chi2       =     0.0000
```

cases	IRR	Std. Err.	z	P>|z|	[95% Conf.	Interval]
mass4						
1	1.185258	.1176035	1.71	0.087	.9757871	1.439696
2	.9096095	.1069167	-0.81	0.420	.7224431	1.145266
3	.7401525	.1030979	-2.16	0.031	.5633198	.9724951
4	.6979264	.112031	-2.24	0.025	.5095362	.9559701
5	.5606689	.1017057	-3.19	0.001	.3929144	.8000461
6	.4650396	.0938509	-3.79	0.000	.313117	.6906741
7	.3496517	.0782323	-4.70	0.000	.2255198	.542109
8	.3327507	.0814023	-4.50	0.000	.2060088	.5374675
9	.3380323	.0910834	-4.03	0.000	.1993428	.5732128
10	.2857476	.0835348	-4.28	0.000	.1611181	.5067817
11	.2210622	.0707774	-4.71	0.000	.1180282	.414041
12	.210928	.0782178	-4.20	0.000	.1019732	.4362974
13	.1535516	.0684175	-4.21	0.000	.0641191	.3677234
14	.0618684	.0348283	-4.94	0.000	.0205255	.1864855
business4						
1	1.196626	.1353321	1.59	0.112	.9587203	1.493567
2	.9023284	.126786	-0.73	0.465	.6851133	1.188411
3	.789744	.1262493	-1.48	0.140	.5773143	1.08034
4	.6610521	.1180563	-2.32	0.020	.465823	.9381029
5	.5675012	.1113741	-2.89	0.004	.3862918	.833716
6	.583267	.1282284	-2.45	0.014	.3790835	.8974285
7	.4548825	.1127513	-3.18	0.001	.279842	.7394106
8	.4323347	.1172199	-3.09	0.002	.2541159	.7355433
9	.2882276	.0863427	-4.15	0.000	.1602308	.5184719
10	.2190637	.073024	-4.56	0.000	.1139792	.4210319
11	.1651703	.0647106	-4.60	0.000	.0766385	.3559727
12	.0091631	.0104551	-4.11	0.000	.0009791	.0857558
education4						
1	1.456755	.1490419	3.68	0.000	1.192064	1.780221
2	1.54878	.2073416	3.27	0.001	1.19134	2.013462
3	1.457869	.2329389	2.36	0.018	1.065892	1.993994
4	1.092813	.2020773	0.48	0.631	.7605824	1.570167
5	.8684146	.1800988	-0.68	0.496	.5783598	1.303936
6	.5471565	.1261643	-2.62	0.009	.348209	.8597716
7	.3983053	.1022937	-3.58	0.000	.2407734	.6589064
8	.2216427	.0626413	-5.33	0.000	.1273751	.385676
9	.1645677	.0512526	-5.79	0.000	.0893816	.3029988
10	.1116608	.0379659	-6.45	0.000	.0573433	.2174296
11	.0856576	.0331237	-6.35	0.000	.0401427	.1827785
12	.0908273	.0582404	-3.74	0.000	.0258469	.3191722
13	.0548464	.0509094	-3.13	0.002	.008893	.338258
non_essential4						
1	1.139119	.1308484	1.13	0.257	.9094802	1.42674
2	1.258193	.1638699	1.76	0.078	.9747309	1.624088
3	1.144833	.1587013	0.98	0.329	.8724596	1.502239
4	1.017415	.1440874	0.12	0.903	.7708147	1.342908
5	1.047637	.1545559	0.32	0.752	.7845749	1.398901
6	.8795601	.1447269	-0.78	0.435	.637096	1.2143
7	.8038324	.1438234	-1.22	0.222	.5660656	1.141469
8	.7328725	.1437931	-1.58	0.113	.4989059	1.07656
9	1.067588	.2528525	0.28	0.782	.6711214	1.698268
10	1.020293	.3168747	0.06	0.948	.5550926	1.875359
11	2.773997	1.677676	1.69	0.092	.8478341	9.07614
home4						
1	1.050294	.1224825	0.42	0.674	.8356919	1.320006

```
                  2 |   1.442408   .1781364     2.97   0.003      1.13231    1.837431
                  3 |    2.18335   .2813717     6.06   0.000     1.696007    2.810729
                  4 |   2.491129   .3259727     6.98   0.000     1.927585    3.219428
                  5 |   2.716038   .3724418     7.29   0.000     2.075932    3.553517
                  6 |    2.71914   .4031361     6.75   0.000      2.03345    3.636048
                  7 |   3.307177   .5339675     7.41   0.000     2.410043    4.538267
                  8 |   2.940677   .5413366     5.86   0.000     2.049997    4.218338
                  9 |   2.660378   .6168096     4.22   0.000     1.688853    4.190779
                 10 |   2.581587   .8114433     3.02   0.003      1.39424     4.78009
                 11 |   .8914859   .7986507    -0.13   0.898     .1540115     5.16031
                    |
             masks4 |
                  1 |    .655428   .0705672    -3.92   0.000     .5307375    .8094132
                  2 |   .5767203   .0670066    -4.74   0.000     .4592697    .7242068
                  3 |   .5849548   .0734933    -4.27   0.000     .4572755    .7482843
                  4 |   .4436336   .0613972    -5.87   0.000     .3382371     .581872
                  5 |   .6561941   .1000257    -2.76   0.006     .4867221    .8846746
                  6 |   .6569734   .1262464    -2.19   0.029     .4507937    .9574536
                  7 |   .7840156   .2008676    -0.95   0.342     .4745083    1.295405
                  8 |   .8409724   .2470652    -0.59   0.556      .472837    1.495726
                  9 |   1.362676   .4150369     1.02   0.310     .7501322     2.47541
                 10 |   1.381992   .6027965     0.74   0.458     .5877992    3.249243
                 11 |   2.888456   2.179965     1.41   0.160     .6580364     12.6789
                    |
       dayfromfirst |   1.172339   .0085949    21.69   0.000     1.155614    1.189306
    tests1mpop16apr |   1.000052   .0000104     5.06   0.000     1.000032    1.000073
              _cons |   2.54e-07   5.24e-08   -73.46   0.000     1.69e-07    3.80e-07
ln(popdata2018)     |          1  (exposure)
--------------------+----------------------------------------------------------------
           /lnalpha |   -.816131   .0384955                     -.8915807   -.7406813
--------------------+----------------------------------------------------------------
country_code        |
          var(_cons)|   .3986612   .1128118                      .2289464    .6941832
-------------------------------------------------------------------------------------
Note: Estimates are transformed only in the first equation.
Note: _cons estimates baseline incidence rate (conditional on zero random effects).
LR test vs. nbinomial model: chibar2(01) = 486.54     Prob >= chibar2 = 0.0000
```

```
3 . menbreg cases ib0.mass10 ib0.business10 ib0.education10 ib0.non_essential10 ib0.home10 ib0.masks10 dayfromfirst te

Fitting fixed-effects model:

Iteration 0:   log likelihood = -9994.7123  (not concave)
Iteration 1:   log likelihood = -9729.7216
Iteration 2:   log likelihood = -9400.1142
Iteration 3:   log likelihood = -9314.0225
Iteration 4:   log likelihood = -9310.3497
Iteration 5:   log likelihood =  -9310.341
Iteration 6:   log likelihood =  -9310.341

Refining starting values:

Grid node 0:   log likelihood = -9172.6869

Fitting full model:

Iteration 0:   log likelihood = -9172.6869  (not concave)
Iteration 1:   log likelihood = -9166.0565  (not concave)
Iteration 2:   log likelihood = -9159.8536
Iteration 3:   log likelihood = -9116.4445
Iteration 4:   log likelihood = -9108.8947
Iteration 5:   log likelihood = -9108.8151
Iteration 6:   log likelihood = -9108.8147

Mixed-effects nbinomial regression              Number of obs     =      1,588
Overdispersion:            mean
Group variable:     country_code                Number of groups  =         30
```

```
                                                Obs per group:
                                                             min =         42
                                                             avg =       52.9
                                                             max =         63

Integration method: mvaghermite                 Integration pts.  =          7

                                                Wald chi2(33)     =    3378.08
Log likelihood = -9108.8147                     Prob > chi2       =     0.0000
```

cases	IRR	Std. Err.	z	P>|z|	[95% Conf.	Interval]
mass10						
1	1.344834	.1218836	3.27	0.001	1.125962	1.606253
2	1.142497	.1495364	1.02	0.309	.8839848	1.476607
3	.8847125	.1507808	-0.72	0.472	.6334797	1.235582
4	.6812934	.1465837	-1.78	0.074	.4468831	1.038662
5	.4624673	.1235176	-2.89	0.004	.2739926	.7805906
6	.1330454	.0568681	-4.72	0.000	.0575662	.307491
business10						
1	1.242759	.1301701	2.07	0.038	1.012115	1.525964
2	.9461983	.1383282	-0.38	0.705	.7104637	1.26015
3	.788907	.148699	-1.26	0.208	.5452367	1.141475
4	.5075358	.1206429	-2.85	0.004	.3185174	.8087236
5	.3524099	.1169214	-3.14	0.002	.1839244	.675238
education10						
1	1.480886	.1430543	4.06	0.000	1.225448	1.789567
2	1.165597	.1648493	1.08	0.279	.8834126	1.537917
3	.5976606	.1088434	-2.83	0.005	.4182514	.8540274
4	.3167193	.0719422	-5.06	0.000	.2029204	.4943373
5	.211947	.0606602	-5.42	0.000	.120951	.3714027
non_essential10						
1	1.121458	.1166766	1.10	0.271	.9145844	1.375124
2	1.05786	.1320182	0.45	0.652	.8283241	1.351001
3	.9092745	.1379363	-0.63	0.531	.6754113	1.224113
4	1.167886	.2336179	0.78	0.438	.7890969	1.728504
5	3.964275	1.980284	2.76	0.006	1.489227	10.55277
home10						
1	1.273257	.1249543	2.46	0.014	1.050464	1.543302
2	1.982338	.2200223	6.17	0.000	1.594783	2.464073
3	1.872801	.2487633	4.72	0.000	1.443534	2.429721
4	1.56168	.285629	2.44	0.015	1.09121	2.23499
5	.4451837	.2615056	-1.38	0.168	.1407779	1.40781
masks10						
1	.5949662	.0519296	-5.95	0.000	.5014158	.7059705
2	.4794569	.052917	-6.66	0.000	.3861924	.5952446
3	.4821065	.0823776	-4.27	0.000	.3449039	.6738882
4	.7418492	.1820478	-1.22	0.224	.458599	1.200047
5	1.567337	1.186874	0.59	0.553	.3552924	6.914153
dayfromfirst	1.123644	.0068083	19.24	0.000	1.110379	1.137067
tests1mpop16apr	1.000054	8.33e-06	6.43	0.000	1.000037	1.00007
_cons	3.34e-07	5.66e-08	-87.86	0.000	2.39e-07	4.65e-07
ln(popdata2018)	1	(exposure)				
/lnalpha	-.6825385	.0376508			-.7563328	-.6087442
**country_code**						
var(_cons)	.2505226	.0693896			.1455732	.4311339

Note: Estimates are transformed only in the first equation.
Note: **_cons** estimates baseline incidence rate (conditional on zero random effects).
LR test vs. nbinomial model: chibar2(01) = **403.05**   Prob >= chibar2 = **0.0000**

```
4 . testparm ib0.mass10

 ( 1)  [cases]1.mass10 = 0
 ( 2)  [cases]2.mass10 = 0
 ( 3)  [cases]3.mass10 = 0
 ( 4)  [cases]4.mass10 = 0
 ( 5)  [cases]5.mass10 = 0
 ( 6)  [cases]6.mass10 = 0

           chi2(  6) =   64.89
         Prob > chi2 =    0.0000

5 . menbreg cases ib0.mass14 ib0.business14 ib0.education14 ib0.non_essential14 ib0.home14 ib0.masks14 dayfromfirst te
  note: 4.home14 omitted because of collinearity

  Fitting fixed-effects model:

  Iteration 0:   log likelihood = -10004.262  (not concave)
  Iteration 1:   log likelihood = -9743.3976
  Iteration 2:   log likelihood =  -9461.711
  Iteration 3:   log likelihood = -9345.9307
  Iteration 4:   log likelihood = -9345.2049
  Iteration 5:   log likelihood = -9345.2044

  Refining starting values:

  Grid node 0:   log likelihood = -9214.5766

  Fitting full model:

  Iteration 0:   log likelihood = -9214.5766  (not concave)
  Iteration 1:   log likelihood = -9207.8849  (not concave)
  Iteration 2:   log likelihood = -9201.7211
  Iteration 3:   log likelihood = -9168.2085
  Iteration 4:   log likelihood = -9162.6838
  Iteration 5:   log likelihood = -9161.9602
  Iteration 6:   log likelihood = -9161.9209
  Iteration 7:   log likelihood = -9161.9208

  Mixed-effects nbinomial regression              Number of obs     =      1,588
  Overdispersion:            mean
  Group variable:    country_code                 Number of groups  =         30

                                                  Obs per group:
                                                                min =         42
                                                                avg =       52.9
                                                                max =         63

  Integration method: mvaghermite                 Integration pts.  =          7

                                                  Wald chi2(24)     =    3035.84
  Log likelihood = -9161.9208                     Prob > chi2       =     0.0000
```

cases	IRR	Std. Err.	z	P>|z|	[95% Conf.	Interval]
mass14						
1	1.546279	.1388527	4.85	0.000	1.296736	1.843843
2	1.44411	.194042	2.73	0.006	1.109752	1.879206
3	1.202014	.2197375	1.01	0.314	.8400459	1.719949
4	.8038274	.1994619	-0.88	0.379	.4942486	1.307315
business14						
1	1.204076	.12594	1.78	0.076	.9808952	1.478036
2	.9057518	.1367136	-0.66	0.512	.6737975	1.217556
3	.6316922	.1282855	-2.26	0.024	.4242692	.9405231
4	.3694655	.15298	-2.40	0.016	.1641071	.8318027
education14						
1	1.565542	.1509728	4.65	0.000	1.295923	1.891255
2	1.037304	.15069	0.25	0.801	.7802814	1.378989
3	.4584379	.0886586	-4.03	0.000	.3138082	.6697253
4	.3896325	.1151281	-3.19	0.001	.218345	.6952919

```
                 |
non_essential14  |
              1  |   1.090845   .1123048     0.84   0.398     .8915174    1.334738
              2  |   .9366864   .1212635    -0.51   0.613     .7267709    1.207232
              3  |    .811321   .1463667    -1.16   0.246     .5696806    1.155458
              4  |   1.809959   1.183796     0.91   0.364     .5022778     6.52219
                 |
         home14  |
              1  |   1.478777   .1440292     4.02   0.000     1.221793    1.789812
              2  |   1.825602   .2120113     5.18   0.000     1.453965    2.292229
              3  |   1.762915    .287684     3.47   0.001     1.280344     2.42737
              4  |          1  (omitted)
                 |
        masks14  |
              1  |   .5638435   .0473464    -6.82   0.000     .4782803    .6647138
              2  |   .4510717   .0561698    -6.39   0.000     .3533861    .5757601
              3  |   .7172957   .1678056    -1.42   0.156     .4534887    1.134567
                 |
   dayfromfirst  |   1.098089   .0058299    17.62   0.000     1.086722    1.109576
tests1mpop16apr  |   1.000056   7.86e-06     7.09   0.000      1.00004    1.000071
          _cons  |   3.78e-07   6.11e-08   -91.54   0.000     2.75e-07    5.19e-07
ln(popdata2018)  |          1  (exposure)
-----------------+----------------------------------------------------------------
       /lnalpha  |  -.6096359   .0371429                     -.6824347   -.5368371
-----------------+----------------------------------------------------------------
country_code     |
      var(_cons) |   .2206711   .0606155                      .1288046    .3780588
----------------------------------------------------------------------------------
Note: Estimates are transformed only in the first equation.
Note: _cons estimates baseline incidence rate (conditional on zero random effects).
LR test vs. nbinomial model: chibar2(01) = 366.57     Prob >= chibar2 = 0.0000
```

```
6 . menbreg deaths ib0.mass ib0.business ib0.education ib0.non_essential ib0.home ib0.masks dayfromfirst tests1mpop16a

Fitting fixed-effects model:

Iteration 0:   log likelihood = -6092.0787  (not concave)
Iteration 1:   log likelihood =  -5450.907  (not concave)
Iteration 2:   log likelihood = -5093.0957  (not concave)
Iteration 3:   log likelihood = -5002.9839
Iteration 4:   log likelihood =  -4827.041  (backed up)
Iteration 5:   log likelihood = -4626.4817
Iteration 6:   log likelihood = -4620.6277
Iteration 7:   log likelihood =  -4620.566
Iteration 8:   log likelihood =  -4620.566

Refining starting values:

Grid node 0:   log likelihood = -4349.6314

Fitting full model:

Iteration 0:   log likelihood = -4349.6314
Iteration 1:   log likelihood = -4132.8538
Iteration 2:   log likelihood = -4098.1002
Iteration 3:   log likelihood = -4096.2717
Iteration 4:   log likelihood = -4096.2635
Iteration 5:   log likelihood = -4096.2635

Mixed-effects nbinomial regression              Number of obs     =      1,588
Overdispersion:             mean
Group variable:     country_code                Number of groups  =         30

                                                Obs per group:
                                                              min =         42
                                                              avg =       52.9
                                                              max =         63

Integration method: mvaghermite                 Integration pts.  =          7
```

Wald chi2(38) = **4664.97**
Log likelihood = **-4096.2635**   Prob > chi2 = **0.0000**

deaths	IRR	Std. Err.	z	P>|z|	[95% Conf.	Interval]
mass						
1	.7563676	.120079	-1.76	0.079	.5541129	1.032446
2	.5832463	.1076164	-2.92	0.003	.406251	.8373551
3	.5948864	.132347	-2.33	0.020	.384648	.9200357
4	.5578361	.1458002	-2.23	0.026	.3342183	.9310714
5	.5042393	.1518256	-2.27	0.023	.2794739	.9097709
6	.4930222	.1732332	-2.01	0.044	.2476167	.9816419
business						
1	1.066555	.1597097	0.43	0.667	.7952827	1.43036
2	1.073205	.19744	0.38	0.701	.7483161	1.539147
3	.7191153	.1582015	-1.50	0.134	.4672387	1.106772
4	.4951598	.130937	-2.66	0.008	.2948892	.8314419
5	.415128	.1298009	-2.81	0.005	.2249216	.7661838
6	.3742309	.1370291	-2.68	0.007	.1825836	.7670388
education						
1	2.509827	.3650106	6.33	0.000	1.887345	3.337615
2	3.140665	.6171348	5.82	0.000	2.136791	4.616163
3	2.756948	.6459132	4.33	0.000	1.741824	4.363679
4	2.019311	.5457603	2.60	0.009	1.188909	3.429714
5	1.096814	.3385769	0.30	0.765	.5989282	2.008591
6	.5517744	.1932168	-1.70	0.089	.277775	1.096049
non_essential						
1	1.396027	.2180175	2.14	0.033	1.027926	1.895946
2	1.405768	.2414743	1.98	0.047	1.003921	1.968466
3	1.418574	.2602883	1.91	0.057	.9900763	2.032523
4	1.440418	.3027939	1.74	0.083	.9540155	2.174812
5	1.044527	.2531821	0.18	0.857	.649528	1.679739
6	.7685437	.2336021	-0.87	0.386	.4235866	1.394424
home						
1	1.304813	.2010532	1.73	0.084	.964695	1.764844
2	2.007811	.3310027	4.23	0.000	1.453436	2.773638
3	2.2306	.3901335	4.59	0.000	1.583237	3.14266
4	1.985532	.3800538	3.58	0.000	1.364417	2.889393
5	1.835626	.4052875	2.75	0.006	1.190824	2.829573
6	1.213501	.3408576	0.69	0.491	.6997581	2.104419
masks						
1	.9091138	.0910322	-0.95	0.341	.7471108	1.106246
2	.8940483	.103534	-0.97	0.333	.7125065	1.121846
3	.9701593	.1395542	-0.21	0.833	.7318137	1.286132
4	1.399045	.3072944	1.53	0.126	.9096386	2.151764
5	1.357023	.4367027	0.95	0.343	.7222082	2.549833
6	1.453493	.6602568	0.82	0.410	.5966917	3.540591
dayfromfirst	1.167611	.0100284	18.04	0.000	1.14812	1.187433
tests1mpop16apr	1.000021	.0000182	1.17	0.243	.9999855	1.000057
_cons	2.98e-09	1.07e-09	-54.76	0.000	1.47e-09	6.01e-09
ln(popdata2018)	1	(exposure)				
/lnalpha	-1.205326	.0668716			-1.336392	-1.07426
**country_code**						
var(_cons)	1.190973	.3250558			.6975593	2.033401

Note: Estimates are transformed only in the first equation.
Note: **_cons** estimates baseline incidence rate (conditional on zero random effects).
LR test vs. nbinomial model: chibar2(01) = **1048.60**   Prob >= chibar2 = **0.0000**

```
7 . menbreg deaths ib0.mass4 ib0.business4 ib0.education4 ib0.non_essential4 ib0.home4 ib0.masks4 dayfromfirst tests1m

Fitting fixed-effects model:

Iteration 0:   log likelihood = -6085.9998  (not concave)
Iteration 1:   log likelihood = -5456.6922  (not concave)
Iteration 2:   log likelihood = -5087.2181  (not concave)
Iteration 3:   log likelihood = -4993.0782  (not concave)
Iteration 4:   log likelihood = -4947.6529
Iteration 5:   log likelihood =   -4649.61
Iteration 6:   log likelihood = -4596.5313
Iteration 7:   log likelihood = -4595.8369
Iteration 8:   log likelihood = -4595.8358
Iteration 9:   log likelihood = -4595.8358

Refining starting values:

Grid node 0:   log likelihood = -4329.3104

Fitting full model:

Iteration 0:   log likelihood = -4329.3104
Iteration 1:   log likelihood = -4114.8771
Iteration 2:   log likelihood = -4062.0213
Iteration 3:   log likelihood = -4048.4743
Iteration 4:   log likelihood = -4048.4537
Iteration 5:   log likelihood = -4048.4537

Mixed-effects nbinomial regression              Number of obs     =      1,588
Overdispersion:            mean
Group variable:    country_code                 Number of groups  =         30

                                                Obs per group:
                                                              min =         42
                                                              avg =       52.9
                                                              max =         63

Integration method: mvaghermite                 Integration pts.  =          7

                                                Wald chi2(74)     =    4914.42
Log likelihood = -4048.4537                     Prob > chi2       =     0.0000
```

deaths	IRR	Std. Err.	z	P>|z|	[95% Conf.	Interval]
mass4						
1	.5847977	.1084222	-2.89	0.004	.4066229	.8410455
2	.5879855	.1128876	-2.77	0.006	.4035934	.8566218
3	.3668961	.0810156	-4.54	0.000	.2380051	.5655876
4	.3894459	.0994524	-3.69	0.000	.2360895	.642418
5	.3189652	.0926378	-3.93	0.000	.1805206	.5635855
6	.2822302	.0922415	-3.87	0.000	.1487321	.5355529
7	.2450735	.0896624	-3.84	0.000	.1196401	.5020144
8	.2146618	.0865236	-3.82	0.000	.097423	.4729859
9	.1879309	.0837082	-3.75	0.000	.0784974	.4499255
10	.1686319	.0816859	-3.67	0.000	.0652551	.4357775
11	.1707839	.0900275	-3.35	0.001	.0607774	.4799008
12	.1472727	.0855079	-3.30	0.001	.0471962	.4595552
13	.1240041	.0795963	-3.25	0.001	.0352422	.4363239
14	.0943584	.071085	-3.13	0.002	.021554	.4130801
business4						
1	1.097531	.1790413	0.57	0.568	.7971858	1.511034
2	.9655254	.1806591	-0.19	0.851	.6691071	1.393259
3	1.130661	.2418595	0.57	0.566	.7434505	1.719542
4	.8317088	.2021432	-0.76	0.448	.516524	1.339221
5	.6129191	.1689121	-1.78	0.076	.3571278	1.05192
6	.5121274	.1606383	-2.13	0.033	.2769382	.9470506
7	.4135227	.1467851	-2.49	0.013	.2062313	.8291708
8	.3753815	.1481539	-2.48	0.013	.1731907	.8136194
9	.3705288	.1619581	-2.27	0.023	.157312	.8727343
10	.3681416	.1775462	-2.07	0.038	.143054	.9473923
11	.3111824	.1710421	-2.12	0.034	.105962	.9138603

12	.1111157	.1535649	-1.59	0.112	.0074027	1.667859
education4						
1	2.056265	.3068929	4.83	0.000	1.534756	2.754984
2	2.623156	.4945699	5.11	0.000	1.812753	3.795856
3	2.668537	.6027761	4.35	0.000	1.713964	4.15475
4	2.425121	.6178535	3.48	0.001	1.471875	3.995728
5	2.349112	.6718227	2.99	0.003	1.341123	4.114707
6	1.985382	.6332169	2.15	0.032	1.062582	3.70959
7	1.367885	.4844265	0.88	0.376	.6832864	2.738397
8	.8678009	.3380697	-0.36	0.716	.4044054	1.862186
9	.609483	.2606812	-1.16	0.247	.2635696	1.409379
10	.3384913	.15844	-2.31	0.021	.1352445	.8471793
11	.2831938	.1458306	-2.45	0.014	.1032185	.7769802
12	.1266484	.0854567	-3.06	0.002	.0337479	.475283
13	.0735043	.062757	-3.06	0.002	.0137901	.3917934
non_essential4						
1	1.378051	.229698	1.92	0.054	.9939952	1.910497
2	1.334065	.2391555	1.61	0.108	.9388226	1.895704
3	1.300853	.2380027	1.44	0.151	.9088523	1.861929
4	1.325037	.2503383	1.49	0.136	.914979	1.918867
5	1.326435	.2586255	1.45	0.147	.9051498	1.943799
6	1.41912	.3067426	1.62	0.105	.9290346	2.167735
7	1.314033	.3094178	1.16	0.246	.8282709	2.084684
8	1.058531	.2738149	0.22	0.826	.6375577	1.757469
9	.9468984	.2855925	-0.18	0.856	.5242927	1.710145
10	.681326	.2583866	-1.01	0.312	.3239997	1.432733
11	1.030781	.6757346	0.05	0.963	.2852047	3.725431
home4						
1	1.301498	.2272646	1.51	0.131	.9242905	1.832645
2	1.547739	.2765779	2.44	0.015	1.090409	2.196877
3	2.382469	.4240976	4.88	0.000	1.680765	3.377129
4	2.368919	.4322144	4.73	0.000	1.65671	3.3873
5	2.575174	.4875535	5.00	0.000	1.776847	3.732185
6	2.409067	.4857608	4.36	0.000	1.622609	3.576712
7	2.317956	.5088787	3.83	0.000	1.50742	3.564314
8	2.19568	.5343065	3.23	0.001	1.362806	3.537563
9	1.783916	.5160842	2.00	0.045	1.011866	3.145037
10	1.516464	.5632429	1.12	0.262	.7322846	3.140394
11	.8600512	.7007593	-0.19	0.853	.1741709	4.24691
masks4						
1	.9292004	.1070975	-0.64	0.524	.7413135	1.164708
2	.9410561	.1158773	-0.49	0.622	.739269	1.197922
3	.9575459	.1279443	-0.32	0.745	.7369274	1.244212
4	1.024722	.1585697	0.16	0.875	.7566373	1.387793
5	1.11171	.1904436	0.62	0.536	.7946477	1.555278
6	1.590809	.3522862	2.10	0.036	1.030667	2.455372
7	1.490327	.4674364	1.27	0.203	.8059439	2.755867
8	1.038825	.4130926	0.10	0.924	.4764953	2.264779
9	3.151694	1.254835	2.88	0.004	1.444247	6.877754
10	2.579486	1.956182	1.25	0.211	.583465	11.40385
11	2.780446	3.416485	0.83	0.405	.2501477	30.90524
dayfromfirst	1.206331	.01368	16.54	0.000	1.179815	1.233444
tests1mpop16apr	1.000018	.0000192	0.92	0.357	.99998	1.000055
_cons	2.61e-09	9.77e-10	-52.74	0.000	1.25e-09	5.43e-09
ln(popdata2018)	1	(exposure)				
/lnalpha	-1.326459	.0685182			-1.460752	-1.192165
**country_code**						
var(_cons)	1.306631	.3616677			.7595306	2.247814

Note: Estimates are transformed only in the first equation.
Note: **_cons** estimates baseline incidence rate (conditional on zero random effects).
LR test vs. nbinomial model: chibar2(01) = **1094.76** Prob >= chibar2 = **0.0000**

```
8 . menbreg deaths ib0.mass10 ib0.business10 ib0.education10 ib0.non_essential10 ib0.home10 ib0.masks10 dayfromfirst t

Fitting fixed-effects model:

Iteration 0:   log likelihood = -6096.7024  (not concave)
Iteration 1:   log likelihood = -5442.2489  (not concave)
Iteration 2:   log likelihood = -5099.6838  (not concave)
Iteration 3:   log likelihood = -5013.7263
Iteration 4:   log likelihood = -4717.0454  (backed up)
Iteration 5:   log likelihood = -4649.6372
Iteration 6:   log likelihood = -4648.6644
Iteration 7:   log likelihood =   -4648.66
Iteration 8:   log likelihood =   -4648.66

Refining starting values:

Grid node 0:   log likelihood = -4387.6526

Fitting full model:

Iteration 0:   log likelihood = -4387.6526
Iteration 1:   log likelihood =  -4188.023
Iteration 2:   log likelihood = -4148.1391
Iteration 3:   log likelihood = -4146.1946
Iteration 4:   log likelihood = -4146.1839
Iteration 5:   log likelihood = -4146.1839

Mixed-effects nbinomial regression              Number of obs     =      1,588
Overdispersion:             mean
Group variable:     country_code                Number of groups  =         30

                                                Obs per group:
                                                              min =         42
                                                              avg =       52.9
                                                              max =         63

Integration method: mvaghermite                 Integration pts.  =          7

                                                Wald chi2(33)     =    4358.84
Log likelihood = -4146.1839                     Prob > chi2       =     0.0000
```

deaths	IRR	Std. Err.	z	P>|z|	[95% Conf.	Interval]
mass10						
1	.7933842	.1263504	-1.45	0.146	.5806648	1.084031
2	.6462756	.1314059	-2.15	0.032	.4338551	.9626996
3	.6323483	.1586684	-1.83	0.068	.3866993	1.034045
4	.4859425	.1477044	-2.37	0.018	.2678295	.8816809
5	.3990637	.1444065	-2.54	0.011	.1963468	.8110744
6	.1616749	.0796189	-3.70	0.000	.0615826	.4244512
business10						
1	1.354599	.202174	2.03	0.042	1.011041	1.8149
2	1.20092	.2335491	0.94	0.346	.8203075	1.758133
3	.8332331	.2080098	-0.73	0.465	.5108237	1.359133
4	.7346226	.2298941	-0.99	0.324	.397821	1.356566
5	.6529924	.2649127	-1.05	0.293	.2948383	1.446213
education10						
1	2.618531	.3908756	6.45	0.000	1.954323	3.508479
2	3.272596	.651684	5.95	0.000	2.21508	4.834986
3	2.401223	.5825608	3.61	0.000	1.492528	3.863158
4	1.280701	.372342	0.85	0.395	.7243953	2.264228
5	.8868284	.3091561	-0.34	0.730	.4478198	1.756207
non_essential10						
1	1.304915	.2045242	1.70	0.090	.9597769	1.774166
2	1.37905	.2423596	1.83	0.067	.9772074	1.946138
3	1.455649	.3032486	1.80	0.072	.9676759	2.189695
4	1.271133	.324759	0.94	0.348	.7704057	2.09731
5	1.779882	.9400789	1.09	0.275	.6321393	5.011522

home10						
1	1.487145	.2242743	2.63	0.009	1.106585	1.998581
2	1.876879	.313318	3.77	0.000	1.353133	2.603348
3	1.403147	.2671175	1.78	0.075	.9661836	2.03773
4	.8840621	.208023	-0.52	0.600	.5574321	1.402083
5	.1816888	.1060076	-2.92	0.003	.0579013	.570122
masks10						
1	.8454197	.0820308	-1.73	0.084	.6990054	1.022502
2	.8645704	.1104758	-1.14	0.255	.6730275	1.110626
3	1.043792	.2166648	0.21	0.836	.6949062	1.56784
4	1.230409	.4119592	0.62	0.536	.6383439	2.371616
5	1.272183	1.557947	0.20	0.844	.1153845	14.02659
dayfromfirst	1.147815	.0090551	17.48	0.000	1.130204	1.165701
tests1mpop16apr	1.000016	.000019	0.82	0.410	.9999784	1.000053
_cons	3.40e-09	1.26e-09	-52.51	0.000	1.64e-09	7.04e-09
ln(popdata2018)	1	(exposure)				
/lnalpha	-1.085879	.065591			-1.214435	-.957323
**country_code**						
var(_cons)	1.294418	.3538466			.7575077	2.211882

```
Note: Estimates are transformed only in the first equation.
Note: _cons estimates baseline incidence rate (conditional on zero random effects).
LR test vs. nbinomial model: chibar2(01) = 1004.95    Prob >= chibar2 = 0.0000

9 . menbreg deaths ib0.mass14 ib0.business14 ib0.education14 ib0.non_essential14 ib0.home14 ib0.masks14 dayfromfirst t
note: 4.home14 omitted because of collinearity

Fitting fixed-effects model:

Iteration 0:   log likelihood = -6105.2707  (not concave)
Iteration 1:   log likelihood =  -5341.976  (not concave)
Iteration 2:   log likelihood = -5109.2826  (not concave)
Iteration 3:   log likelihood = -5046.7401  (not concave)
Iteration 4:   log likelihood = -4944.9043
Iteration 5:   log likelihood = -4764.9866
Iteration 6:   log likelihood = -4664.3758
Iteration 7:   log likelihood =  -4663.255
Iteration 8:   log likelihood = -4663.2537
Iteration 9:   log likelihood = -4663.2537

Refining starting values:

Grid node 0:   log likelihood = -4394.9043

Fitting full model:

Iteration 0:   log likelihood = -4394.9043
Iteration 1:   log likelihood = -4204.3637
Iteration 2:   log likelihood = -4169.7704
Iteration 3:   log likelihood = -4169.0356
Iteration 4:   log likelihood = -4169.0308
Iteration 5:   log likelihood = -4169.0308

Mixed-effects nbinomial regression             Number of obs     =      1,588
Overdispersion:              mean
Group variable:     country_code               Number of groups  =         30

                                               Obs per group:
                                                             min =         42
                                                             avg =       52.9
                                                             max =         63

Integration method: mvaghermite                Integration pts.  =          7
```

```
                                                Wald chi2(24)     =    4122.31
Log likelihood = -4169.0308                     Prob > chi2       =     0.0000

                 
          deaths        IRR   Std. Err.      z    P>|z|     [95% Conf. Interval]
                 
          mass14  
               1    .8952258   .1423886   -0.70   0.487     .6554601    1.222697
               2    .9902703     .20305   -0.05   0.962     .6625507    1.480091
               3    .8994396   .2289916   -0.42   0.677     .5460866    1.481435
               4    .7018597   .2232273   -1.11   0.266     .3762917    1.309109
                 
      business14  
               1    1.323085   .1970869    1.88   0.060     .9880811     1.77167
               2    .8965988   .1750896   -0.56   0.576     .6114681    1.314687
               3    .6933552   .1757276   -1.44   0.148     .4219128    1.139433
               4    1.083379   .4883515    0.18   0.859      .447803    2.621042
                 
     education14  
               1    2.777188   .4169053    6.80   0.000     2.069307    3.727227
               2    2.789269   .5449698    5.25   0.000     1.901874    4.090712
               3    1.343229    .324296    1.22   0.222     .8368432    2.156034
               4    .5776422   .1929843   -1.64   0.100     .3001105    1.111826
                 
 non_essential14  
               1    1.375352   .2158628    2.03   0.042     1.011153    1.870729
               2    1.356355   .2466374    1.68   0.094      .949713    1.937111
               3    .9347891   .2141295   -0.29   0.768      .596666    1.464522
               4    .3028598   .1800789   -2.01   0.045     .0944325    .9713188
                 
          home14  
               1    1.658136    .249702    3.36   0.001     1.234344    2.227431
               2    1.784602   .3096292    3.34   0.001     1.270157    2.507409
               3    1.438098   .3076567    1.70   0.089     .9455587    2.187201
               4           1  (omitted)
                 
         masks14  
               1    .7787291   .0730946   -2.66   0.008      .647872    .9360168
               2    .7451864   .1077411   -2.03   0.042      .561301    .9893136
               3    .6632024   .2066264   -1.32   0.187     .3601197    1.221364
                 
    dayfromfirst    1.134572   .0075746   18.91   0.000     1.119823    1.149516
tests1mpop16apr      1.00002   .0000182    1.11   0.265     .9999846    1.000056
           _cons    3.38e-09   1.21e-09  -54.49   0.000     1.67e-09    6.81e-09
ln(popdata2018)            1  (exposure)
                 
        /lnalpha   -.9963278   .0634911                     -1.120768   -.8718875
                 
country_code     
       var(_cons)   1.193087    .324013                      .7006589    2.031596
                 
Note: Estimates are transformed only in the first equation.
Note: _cons estimates baseline incidence rate (conditional on zero random effects).
LR test vs. nbinomial model: chibar2(01) = 988.45     Prob >= chibar2 = 0.0000

10 . log close
      name:  <unnamed>
       log:  C:\Users\PaulH\Dropbox\001Covid19 files\Sensitivity analyses with varying time gaps.smcl
  log type:  smcl
 closed on:  28 Feb 2021, 18:07:26
```
